# Genome-wide SNP and microsatellite variation illuminate population-level epidemiology in the *Leishmania donovani* species complex

**DOI:** 10.1016/j.meegid.2011.11.005

**Published:** 2012-01

**Authors:** Tim Downing, Olivia Stark, Manu Vanaerschot, Hideo Imamura, Mandy Sanders, Saskia Decuypere, Simonne de Doncker, Ilse Maes, Suman Rijal, Shyam Sundar, Jean-Claude Dujardin, Matthew Berriman, Gabriele Schönian

**Affiliations:** a Wellcome Trust Sanger Institute, Wellcome Trust Genome Campus, Hinxton CB10 1SA, UK; b Institut für Mikrobiologie und Hygiene, Charité Universitätsmedizin Berlin, Berlin, Germany; c Unit of Molecular Parasitology, Department of Parasitology, Institute of Tropical Medicine, Antwerp, Belgium; d B.P. Koirala Institute of Health Sciences, Ghopa, Dharan, Nepal; e Institute of Medical Sciences, Banaras Hindu University, Varanasi, India

**Keywords:** MLMT, multi-locus microsatellite typing, VL, visceral leishmaniasis, CL, cutaneous leishmaniasis, PKDL, post kala-azar dermal leishmaniasis, SSG, sodium stibogluconate, *Leishmania infantum*, Visceral leishmaniasis, Diversity, Population markers, Population genetics, Drug resistance

## Abstract

The species of the *Leishmania donovani* species complex cause visceral leishmaniasis, a debilitating infectious disease transmitted by sandflies. Understanding molecular changes associated with population structure in these parasites can help unravel their epidemiology and spread in humans. In this study, we used a panel of standard microsatellite loci and genome-wide SNPs to investigate population-level diversity in *L. donovani* strains recently isolated from a small geographic area spanning India, Bihar and Nepal, and compared their variation to that found in diverse strains of the *L. donovani* complex isolates from Europe, Africa and Asia. Microsatellites and SNPs could clearly resolve the phylogenetic relationships of the strains between continents, and microsatellite phylogenies indicated that certain older Indian strains were closely related to African strains. In the context of the anti-malaria spraying campaigns in the 1960s, this was consistent with a pattern of episodic population size contractions and clonal expansions in these parasites that was supported by population history simulations. In sharp contrast to the low resolution provided by microsatellites, SNPs retained a much more fine-scale resolution of population-level variability to the extent that they identified four different lineages from the same region one of which was more closely related to African and European strains than to Indian or Nepalese ones. Joining results of *in vitro* testing the antimonial drug sensitivity with the phylogenetic signals from the SNP data highlighted protein-level mutations revealing a distinct drug-resistant group of Nepalese and Indian *L. donovani*. This study demonstrates the power of genomic data for exploring parasite population structure. Furthermore, markers defining different genetic groups have been discovered that could potentially be applied to investigate drug resistance in clinical *Leishmania* strains.

## Highlights

► Microsatellite typing of *Leishmania donovani* complex isolates discriminates intercontinental groups. ► Genome-wide SNP profiling reveals diversity in a homogeneous population. ► Identification of a novel divergent lineage within a small geographic region. ► SNP-typing of samples resistant and sensitive to treatment drugs.

## 1 Introduction

An estimated 12 million people are currently infected by *Leishmania* parasites, including the visceral (VL), cutaneous (CL) and mucocutaneous forms of the disease (www.who.int/leishmaniasis/). The species of the *Leishmania donovani* complex cause the most severe type of leishmaniasis (VL) in tropical and sub-tropical regions – though asymptomatic infections are common (Rijal et al., 2010). Unlike the other *Leishmania* parasites, those causing VL disseminate to internal organs and are responsible for 50,000 deaths and the loss of over 2.3 million disability-adjusted life years annually (WHO Expert Committee on the Control of the Leishmaniases, 2010). The number of people in India, Nepal and Bangladesh at risk of infection is 190 million and the annual volume of cases in India is over 100,000 – mostly occur in the north-western Indian state of Bihar, adjacent to the Terai region of Nepal, where VL is also endemic (Sundar et al., 2008). This shows the scale of the disease burden and the challenge in combating VL as detailed in the first regional programme of VL elimination in the Indian subcontinent by 2015 (WHO, 2005).

As a result, improved molecular tools are vital for monitoring the epidemiology and diversity of strains in the *L. donovani* species complex. A wide variety of approaches has been applied, including those using multilocus enzyme electrophoresis (Rioux et al., 1990), sequencing of ribosomal loci (Kuhls et al., 2005), microsatellite typing (for example, Ochsenreither et al., 2006), gene sequences (for example, Mauricio et al., 2006), random amplification of polymorphic DNA (Botilde et al., 2006), amplified fragment length polymorphisms (for example, Kumar et al., 2010) and kinetoplastid minicircle DNA (kDNA) restriction fragment length polymorphisms (RFLP; for example, Laurent et al., 2007). Among this multitude of approaches used to assess diversity, kDNA RFLP and multi-locus microsatellite typing (MLMT) have proven to be highly discriminatory for typing *L. donovani* species complex parasites (Botilde et al., 2006). Although kDNA diversity can be informative, the variability in both experimental replication (Bhattarai et al., 2010) and total DNA yield (Downing et al., 2011) limit the effectiveness of this approach. Nonetheless, the hypothesis of multiple origins for drug resistance in these parasites stems from kDNA variation in Nepalese *L. donovani* (Laurent et al., 2007), and was supported by genome-wide variability (Downing et al., 2011).

MLMT has been used extensively for typing strains of the *L. donovani* complex. An initial study investigating variation in 15 microsatellite markers for 91 strains of the *L. donovani* complex from different VL foci in the Mediterranean Basin, East Africa and the Bihar state of India highlighted significant differentiation between the continental groups (Kuhls et al., 2007). However, in contrast to strains from East Africa and the Mediterranean that appeared to be highly variable, Bihari strains had little microsatellite profile diversity. Improved sampling of more and newly isolated strains from Bihar, Nepal and Bangladesh with MLMT suggested that *L. donovani* strains in the Indian subcontinent showed genetic homogeneity regardless of geographical origin, clinical manifestation, and whether they presented *in vitro* or *in vivo* susceptibility to antimonial drugs (Alam et al., 2009). This result was reflected in the poor resolution of MLMT as well as also PCR-RFLP targeting kDNA and genomic coding sequences to clearly determine the genetic relationships of Nepalese strains (Bhattarai et al., 2010). In contrast, wider MLMT analysis of *L. infantum* strains from Europe and South America (*N* = 406), and of East African *L. donovani* (*N* = 123) further supported their considerable diversity (Gelanew et al., 2010; Kuhls et al., 2011).

With ongoing improvements in sequencing technology, genome-wide SNP typing represents a powerful alternative approach for differentiating parasite strains (Mardis, 2011). In this study, we sought to elucidate the variation in the *L. donovani* complex firstly within a population and secondly between species using SNP and microsatellite genotyping, while also comparing the power of each marker type. While both methods were effective at discriminating isolates over long geographic distances, informing on the population history of *Leishmania* strains, microsatellites lacked sufficient power to resolve diversity in closely related strains of Nepalese and Indian *L. donovani*. In contrast, genome-wide SNP variation provided new evolutionary insights into the ongoing diversification of this phenotypically variable set of strains, potential links between the genotypes of the strains with *in vitro* sodium stibogluconate (SSG) resistance, as well as identifying protein sequence mutations that may underlie the phenotypic differences.

## 2 Material and methods

### 2.1 Sample collection

To compare the discriminatory power of microsatellite and SNP typing for unravelling diversity within a set of genetically homogeneous *L. donovani*, 25 clinical isolates taken from a small focus with endemic VL in the Indian subcontinent were examined by assaying their microsatellite and SNP genotypes (Supplementary Fig. 1). These parasites were isolated n the Terai area of Nepal (23) and the nearby Indian state of Bihar (two). Each isolate was independently taken from unique VL patients (with the exception of two strains from BPK173; Rijal et al., 2010). The genomes of 17 of these have been scrutinised (Downing et al., 2011) and so eight of the Nepalese strains represent entirely new samples assessed using both SNPs and microsatellites (Table 1). DNA was isolated and phenotype responses were assessed for *in vitro* susceptibility to SSG relative to the Nepalese reference genome (BPK282/0cl04, see Supplementary data) for lines not already assessed (Rijal et al., 2007; Downing et al., 2011). In total, nine clinical samples were classed as resistant and 15 as sensitive (the phenotype of BPK077/0cl5 was not determined).

Genetic variation between strains of the *L. donovani* complex from different continents was studied using both marker types to frame diversity observed with the Terai-Bihar region. Five additional *L. donovani* strains originally taken in Sri Lanka (L60b), Kenya (LRC-L53, NLB218), Sudan (597LN), Ethiopia (GEBRE1), as well as three *L. infantum* from China (D2) and France (LPN114, LRC-L47) were assessed. All of these caused VL or PKDL except L60b, whose pathology was cutaneous (CL): this strain was closely related to other Sri Lankan ones causing CL (Alam et al., 2009). Genome, enzyme and microsatellite markers have been previously used to classify these eight samples (Kuhls et al., 2005, 2007; Mauricio et al., 2006; Lukes et al., 2007; Zemanová et al., 2007; Kuhls et al., 2008), leading to their usage here as informative representatives of genetic variation in the wider *L. donovani* complex. Ethiopian GEBRE1 and the Kenyan strains LRC-L53 and NLB218 are representatives of East African diversity, though NLB218 may be more divergent within this group (Alam et al., 2009). Among the *L. infantum* samples, French strain LPN114 represents Mediterranean strains belonging to zymodeme MON-1. Strains LRC-L47 (France) and D2 (China) are part of the non-MON-1 group, though D2 has a more divergent genetic profile (Kuhls et al., 2005, 2007).

### 2.2 Microsatellite profiling

In light of the extensive research already performed on microsatellite variability in *L. donovani*, a combination of new and existing microsatellite data yielded a total 193 strains of the *L. donovani* complex that were investigated here. Analysis of variation in tandem repeat numbers at 15 unlinked microsatellites (Supplementary Table 1) was completed for 41 newly typed strains from Bangladesh, the Bihar state of India and Terai region of Nepal (Supplementary Table 2). Their microsatellite profiles were compared to those of 152 strains previously studied of which 25 came from African and European foci endemic for VL (Ochsenreither et al., 2006; Kuhls et al., 2007; Alam et al., 2009; Bhattarai et al., 2010). Of the 193 strains, 168 strains were from the Indian subcontinent, 17 *L. donovani* strains from Sudan (7), Kenya (7), and Ethiopia (3), and eight genetically diverse strains of *L. infantum* were from France (3), China (3), Spain (1) and Tunisia (1). The strains of *L. infantum* represented the MON-1 and different non-MON-1 zymodemes (Ochsenreither et al., 2006; Kuhls et al., 2007).

PCR amplification and determination of the DNA fragment sizes was performed as described elsewhere (Ochsenreither et al., 2006; Kuhls et al., 2007). Ten of the 15 microsatellite markers were invariant in the previously published strains from the Indian subcontinent (Ochsenreither et al., 2006; Kuhls et al., 2007; Alam et al., 2009) and, consequently, five markers (Lm4TA, TubCA, B_Li41-56, F_Li23-41 and CS20) were amplified in the 41 newly typed strains (Supplementary Table 1).

### 2.3 SNP genotyping

SNP variation was examined in the 33 strains (23 Nepalese, two Indian and eight globally diverse *L. donovani* complex strains) out of the 193 strains discussed above. Five Nepalese clones taken from the same patients as three of the 23 Nepalese strains were also analysed to investigate the isolates’ heterogeneity (BPK085/0cl8, BPK085/0cl3, BPK275/0cl3, BPK2880cl7, BPK288/0cl9; see Supplementary Results).

The SNP loci used here were selected from 3549 SNPs discovered in the genomes of 17 strains (Downing et al., 2011). Among this initial dataset, we focused on intermediate-frequency variants to maximise discriminating power. Candidate sites were screened using multiple steps: first, SNPs close to other ones (±200 bp) were excluded, because the amplification process was allele-specific. Second, sites whose adjacent ±100 bp had GC content levels <30% or >70% were excluded. Third, genome-wide BLAST (Basic Local Alignment Search Tool, Altschul et al., 1990) alignments of the candidate regions were used to eliminate non-unique loci (*E*-value < 10^−4^). Fourth, sites were examined using alignments of the *L. donovani* and *infantum* genomes with the Artemis Comparison Tool (Carver et al., 2005) to remove those with evidence of structural variation. A final set of 130 SNPs was obtained, each of which was unique in the *L. donovani* genome: 82 were in coding sequences, of which 51 were nonsynonymous (protein-level) and 31 were synonymous.

Genotyping was conducted in duplicate using mass spectrophotometry of allele-specific amplified DNA on the Sequenom platform (Buetow et al., 2001): SNP site signal detection had a 98.8% success rate (4881/4940). Genomic positions, local sequence, observed genotypes, gene information and GC content of the SNPs are available as a Supplementary file (GenotypingSupplData.xls).

### 2.4 Population genetic analysis of SNP and microsatellite data

In order to develop a comprehensive genetic picture of diversity at the SNP loci, several complementary tools were applied. Analysis of population structure was conducted using a Bayesian population-clustering algorithm to estimate the most likely extent of differentiation between groups of strains independent of a mutation model, and quantified this in an unbiased probabilistic manner to assign strains to discrete populations (Structure v2.3.3; Pritchard et al., 2000). Strains were clustered based on their relative similarity to obtain their probability of membership to each population and inferred ancestry values. The most likely number of populations (*K*) was determined from the likelihood values for each model and their first and second-order rates of change (L(*K*), L(*K*)′, Δ*K*, respectively, Evanno et al., 2005). Admixture modelling assigned strains to clusters and permitted incomplete membership to minimise overfitting (Falush et al., 2003). A burn-in of 10^5^ and run of 2 × 10^6^ steps were used for each simulation conducted in sets of 10 for 1 ⩽ *K* ⩽ 15.

Further elucidation of the SNP-based phylogenetic signals within the cohort was achieved using neighbour-joining trees (Saitou and Nei, 1987) using the maximum composite likelihood method (Tamura et al., 2004) in MEGA v5 (Tamura et al., 2011), as well as median-joining networks (Network v4.2.0.1, Bandelt et al., 1999) and principal component analysis. The greater circle arc distance between the samples’ point of isolation was used to determine the effect of geography on diversity. *F*_ST_ values (Wright, 1951) were determined for each group (Arlequin v2.001, Schneider et al., 2000). Predictions of the functional impact of non-synonymous substitutions on overall protein structure were implemented for SNPs discriminating the major strain subsets for substitutions with significant support (SIFT, http://blocks.fhcrc.org, Ng and Henikoff, 2003; and PolyPhen, http://genetics.bwh.harvard.edu.pph, Ramensky et al., 2002).

In order to compare genetic diversity inferred from both SNPs and microsatellites, parallel analyses were implemented where possible. Consequently, the microsatellite-derived population structure was determined using the same distance-based and Bayesian population-resolution methods. A strain-based genetic distance matrix was calculated based on the proportions of alleles shared between strains (*D*_AS_) based on pairwise distances (Bowcock et al., 1994) with Populations (v1.2.31, http://bioinformatics.org) to resolve the phylogenetic relationships in neighbour-joining trees (MEGA v5).

The Bayesian clustering approach estimated the population structure as outlined above for the SNPs (Structure v2.3.3, Pritchard et al., 2000). L(*K*), L(*K*)′ and Δ*K* were computed from 50 runs for 1 ⩽ *K* ⩽ 15 using a burn-in of 10^4^ and a run length of 10^5^ iterations. The degree of genetic differentiation among the geographic populations was examined using an infinite alleles model for *K* = 3 (Microsatellite Analyser v4.05, Dieringer and Schlötterer, 2003). For each microsatellite, allelic diversity for each population was calculated for the number of individuals, the proportion of polymorphic loci (where the minor allele frequency >0.01), number of alleles, the mean number of alleles, alleles per polymorphic locus, the expected and observed heterozygosity using permutation methods assuming Hardy–Weinberg equilibrium (Genetic Data Analysis v1.0, http://hydrodictyon.eeb.uconn.edu).

To gain an insight into the evolutionary history of the strains studied, we investigated changes in the effective population size (*N*_e_) using Bayesian skyline plots implemented with Beast v1.6.2, Tracer v1.5.0, TreeAnnotator v1.6.2 and Figtree v1.3.1 (Drummond et al., 2005; Drummond and Rambaut, 2007). Posterior density intervals were calculated for the Bihar-Terai strains, both with and without the outgroup strains or divergent strains from the same region, to compute the relative changes in *N*_e_. Time was scaled using a generation length of one day and *N*_e_ was estimated using a mutation rate of 10^−6^ as outlined previously (Leblois et al., 2011).

## 3 Results and discussion

By compiling SNP and microsatellite variation in a set of *L. donovani* strains from a small area of Nepal and India, population genetic patterns of variation were examined in the context of a globally diverse range of strains. In total, 33 strains were assessed using both SNPs (130) and microsatellites (15): these came from Nepal (23), India (2), France (2), Kenya (2), China (1), Ethiopia (1), Sri Lanka (1) and Sudan (1). The contrasting power of these two genetic markers to resolve population-level differentiation emphasizes relative advantages of genome-wide data and well-established approaches in evaluating diversity in *Leishmania* parasites.

### 3.1 Microsatellite population structure in the *L. donovani* complex

Two genetic distance approaches were applied to microsatellite profiles of 193 strains from globally distributed endemic foci. Bayesian clustering of the samples into groups identified three main phylogenetically distinct population clusters (*K* = 3, Supplementary Fig. 2) that appeared to reflect the geographical origins of the strains, consistent with earlier work (Kuhls et al., 2007; Gelanew et al., 2010). Both Bayesian statistics and genetic distance analyses confirmed the major genetic groups that have been previously identified (Alam et al., 2009).

**Fig. 1:**
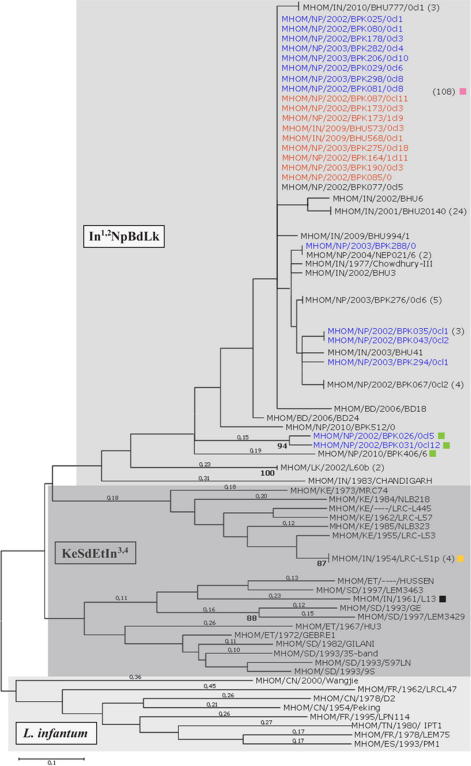
Phylogenetic relationships of *L. donovani* complex strains using microsatellite profiling. A midpoint-rooted neighbour-joining tree constructed from microsatellite data for 193 *L. donovani* complex strains with MEGA using the minimum evolution method. Sub-populations determined with structure (for *K* = 3) are shown as coloured boxes for In^1,2^NpBdLk (grey, top: India, Nepal, Bangladesh and Sri Lanka), KeSdEtIn^3,4^ (dark grey, middle: Kenya, Sudan, Ethiopia and India) and *L. infantum* (pale grey, bottom). Numbers beside samples indicate strains that shared identical microsatellite profiles. One hundred and eight strains had genotype 1a (marked with a pink box); of which those 18 were also SNP genotyped. The most divergent Nepalese strains are marked with green boxes (BPK026/0cl5, BPK031/0cl12, BPK406/6). L60b represents both Sri Lankan strains (the other is L60c). LRC-L51p (orange box) represents two other Indian strains (LRC-L51a and SC23): these grouped with the Kenyan samples. Indian strain L13 (black box) clustered with the Sudanese and Ethiopian strains. Strains subjected to SNP genotyping are highlighted as SSG-resistant (red) and -susceptible (blue). Branch lengths are proportional to the genetic distance: values greater than 0.1 are shown above the branch. Bootstrap values were determined with Populations software for 10^3^ replicates: values greater 87% are shown below the node.

**Fig. 2:**
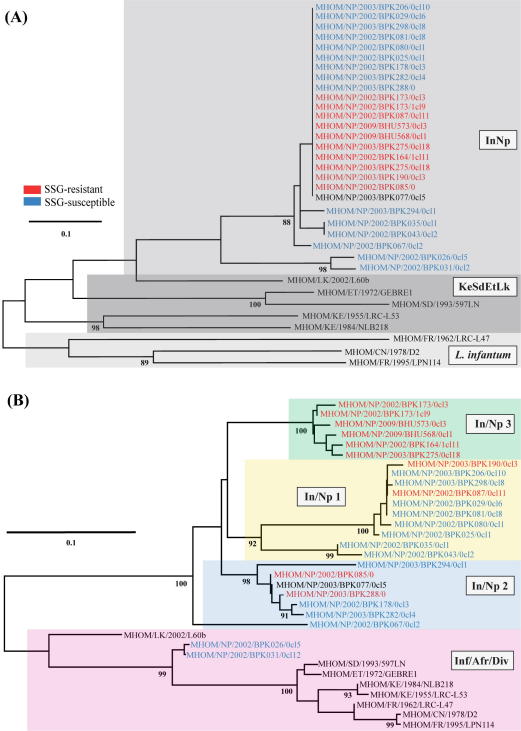
Phylogenetic relationships of strains from the *L. donovani* complex from (A) microsatellite and (B) SNP data. Midpoint-rooted neighbour-joining trees constructed for 33 *L. donovani* complex strains with MEGA using: (A) microsatellite data for *K* = 3 geographical populations called InNp (grey containing Indian – denoted BHU – as well as Nepalese strains named BPK), KeSdEtLk (dark grey, *L. donovani* from Kenya, Sudan, Ethiopia and Sri Lanka) and *L. infantum* (pale grey from China and France) – notably, BPK026/0cl5 and BPK031/0cl12 cluster in the InNp group for microsatellites; and (B) SNP data for *K* = 4 populations denoted In/Np 1 (yellow), 2 (blue) and 3 (green), which were composed of strains from India and Nepal; and Inf/Afr/Div (mauve), which represented Kenyan, Sudanese, Ethiopian, Sri Lankan, Chinese and French *L. donovani* complex strains in addition to two Nepalese samples (BPK026/0cl5 and BPK031/0cl12). Populations were determined using structure for both marker types. *In vitro* responses to SSG are shown as resistant (red) or sensitive (blue). Bootstrap values were determined for 10^4^ (SNPs) or 10^3^ (microsatellites) replicates – only those with more than 87% confidence are shown at each node. Branch lengths are proportional to the genetic distance.

The largest cluster (In^1,2^NpBdLk) consisted of 164 of the 168 strains from the Indian subcontinent (Fig. 1, Supplementary Table 2). This cluster contained 52 strains from Nepal, 88 from India (mainly from Bihar), 21 from Bangladesh, one from the Northwest of India, and two strains from Sri Lanka. Nineteen microsatellite profiles were identified for the strains belonging to In^1,2^NpBdLk of which eight were newly identified in this study. Although 108 strains had an identical microsatellite profile (1a) and most other strains varied at just one marker, there was some differentiation for three Nepalese strains (BPK026/0cl5, BPK031/0cl12 and BPK406/6) within In^1,2^NpBdLk (Fig. 1). The two Sri Lankan strains (L60b and L60c), the only ones in this study isolated from CL cases, and the northwest Indian isolate from Chandigarh appeared as most diverse members of In^1,2^NpBdLk. These three strains shared alleles with strains of both In^1,2^NpBdLk and KeSdEtIn^3,4^, so further sampling is required to clarify their intermediate phylogenetic position (Fig. 2A). Despite moderate variation in these six strains, the general pattern of high genetic homogeneity for In^1,2^NpBdLk was also illustrated by its low observed heterozygosity (0.005) compared to that of KeSdEtIn^3,4^ (0.159; Supplementary Table 3).

The second main population (KeSdEtIn^3,4^) contained four Indian strains (L13, LRC-L51a, LRC-L51p and SC23) genetically distinct from the Nepalese and Bihari samples as well as the strains of *L. donovani* from Sudan, Ethiopia and Kenya included in this study (Fig. 1). These Indian strains were isolated more than 40 years ago and therefore might represent a relic of greater diversity of *L. donovani* existing prior to the 1960s DDT anti-malaria spraying campaigns in the Indian subcontinent (WHO Expert Committee on the Control of the Leishmaniases, 2010). Strains from East Africa were previously shown to belong to two major genetic groups: those from Sudan and North Ethiopia, and also samples from Kenya and South Ethiopia were assigned to different populations, concurring with the existence of different sand fly vectors in the different foci (Kuhls et al., 2007; Gelanew et al., 2010). The same subdivision of population KeSdEtIn^3,4^ was evident here from the population clustering and distance analyses (Fig. 1). One strain isolated from an Indian patient (L13) always grouped with strains from Sudan and Ethiopia whereas the remaining Indian strains (SC23, LRC-L51a, LRC-L51p) clustered with the Kenyan strains essentially as described before (Alam et al., 2009). When the three main geographically defined populations were re-analysed with Structure separately, five genetically distinct sub-populations could be identified that were confirmed by factorial correspondence analysis (Supplementary Fig. 3): this was consistent with previous findings (Alam et al., 2009).

Several patterns emerge from the microsatellite analysis of these strains. The loss of genetic variation within the main Indian cluster might well be a consequence of a population bottleneck related to the DDT spraying efforts. As a result, the population history of the strains studied here from the Indian sub-continent may be composed of a series of periodic expansions from an inferred low number of surviving lineages. This scenario would have been accompanied by a rapid recovery of population size reaching endemic proportions in the main foci in recent decades. Bottlenecks tend to reduce variation at neutral genomic regions like microsatellites, supporting this scenario (Nei et al., 1975). On the other hand, it cannot be excluded that migration of workers between the East Africa and the Indian subcontinent could also have lead to the occurrence of similar profiles in these geographically isolated foci represented by the KeSdEtIn^3,4^ group. However, the four strains from India clustering with the African clades (SC23, LRC-L51a, LRC-L51p, L13; Fig. 1) were all isolated prior or close to strains isolated during the DDT vector elimination program, which favours the bottleneck hypothesis. The number and type of microsatellite alleles appear to trace the phylogenetic history of these Indian samples back to common ancestors within the African cluster, and to some degree for the Sri Lankan and Chandigarh strains.

This lack of variation in samples from Nepal and Bihar was pronounced when compared to the high differentiation observed among other strains sampled in Europe, Asia and Africa. Consequently, although variability among these 15 microsatellites was effective for discriminating geographically distant isolates of the *L. donovani* complex, little genetic resolution of population-level variability for the strains from the Indian subcontinent was found, in spite of the high microsatellite diversity described for strains of *L. donovani* from East Africa and *L. infantum* from the Mediterranean area and South America (Kuhls et al., 2007, 2008, 2011; Gelanew et al., 2010; Leblois et al., 2011).

### 3.2 SNP population structure in the *L. donovani* complex

Strains were assigned to four distinct populations in the set of 33 strains examined at 130 SNPs (Supplementary Table 4). The first of these four to differentiate from the rest was composed of strains from Sudan (1), Ethiopia (1), Kenya (2), Sri Lanka (1), France (2), China (1), and two strains from Nepal (BPK026/0cl5 and BPK031/0cl12). For *K* > 1 population clusters, BPK026/0cl5 and BPK031/0cl12 consistently grouped with these diverse *Leishmania* strains, indicating their higher genetic identity with these rather than the geographically close Nepalese and Bihari strains. Notably, this pair was originally taken from an upland region where VL was not endemic at the time of isolation (Bhattarai et al., 2010).

The second, third and fourth populations (referred to as In/Np 1, 2 and 3) were composed entirely of Nepalese and Indian strains (Fig. 2B). These patterns mirrored the microsatellite data: the Nepalese and Indian strains showed lower diversity in comparison to the other strains. In spite of this, the 130 SNPs used here were sufficient to uniquely resolve 29 of the 33 strains (Supplementary Fig. 4) and presented the novel observation of a divergent lineage (BPK026/0cl5 and BPK031/0cl12). Although the most likely number of groups here suggested a total of five populations (*K* = 5), no strains were assigned to the additional putative group, suggesting that this was not a valid population for this set (Evanno et al., 2005; Hubisz et al., 2009). However, this phantom group was also evident for at *K* = 4, prior to the separate clustering of the In/Np populations 1 and 2, suggesting a wider and deeper clinical sample collection may discover new diversity even in this small geographic area.

The phylogenetic clustering of SSG-resistant and -susceptible strains supported the complex origins of resistance (Supplementary Fig. 5). Furthermore, the absence of any SSG-sensitive strains in In/Np 3 suggested this group could share a recent single origin. There was no evidence of a notable contribution of geography to intra-population SNP diversity as expected, probably a consequence of local host migration and the small region size (Downing et al., 2011). In sharp contrast to the lack of local geographic structure within Nepal and Bihar, the level of differentiation of the three In/Np populations from both the *L. infantum* (*F*_ST_ = 0.64) and combined African and Sri Lankan *L. donovani* (0.56) strains matched that of previous work on European, Asian and African strains (Lukes et al., 2007; Kuhls et al., 2007; Gelanew et al., 2010).

Reconstructing the population history using Bayesian skyline plots provided evidence that the strains from Bihar and Terai have undergone a recent bottleneck followed by a slight increase (Supplementary Fig. 6). This trend was robust to the inclusion of divergent strains, though the magnitude of change in effective population size decreased. Using the calibration of the *L. infantum*–*L. chagasi* split between Europe and South America (Leblois et al., 2011) and the time of isolation of the Nepalese sample (2002–03), the lowest effective population size dates the population recovery for this subset to recently after the Indian parasite elimination programs (1974–84 with 95% limits).

### 3.3 Congruence in population structure between SNPs and microsatellites

**Fig. 3:**
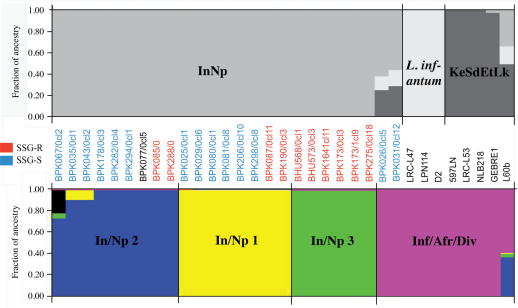
Comparative estimation of population membership for 33 *L. donovani* species complex strains using microsatellite (top) and SNP (bottom) profiling. The assignation probabilities of each strain to populations determined by structure clustering for 33 *L. donovani* samples for: (top) microsatellite loci for *K* = 3 populations shown as InNp in grey (India, named BHU, and Nepal, called BPK), *L. infantum* in pale grey (French and Chinese), and KeSdEtLk in dark grey (Kenyan, Sudanese, Ethiopian and Sri Lankan *L. donovani*); and (bottom) SNP typing for *K* = 5 4 called In/Np 1 in yellow (India and Nepal), In/Np 2 in blue, In/Np 3 in green and Inf/Afr/Div in mauve (BPK026/0cl5 and BPK031/0cl12 from Nepal and Kenyan, Sudanese, Ethiopian, Sri Lankan, Chinese and French *L. donovani* complex strains). Strain colours show the *in vitro* response to SSG exposure as resistant (red) or sensitive (blue). BPK026/0cl5 and BPK031/0cl12 consistently clustered with the global *L. donovani* and *L. infantum* strains for *K* > 1 SNP-ascertained populations, in contrast to their assignation with microsatellites. A fifth hypothetical SNP-based population cluster (shown in black at strain BPK067/0cl2) that had no exclusive ancestry of individual strains suggested the possible circulation of further distinct genetic groups in this small region.

By performing parallel investigations of population structure using the 33 strains that were typed with both SNP and microsatellite markers, the relative capacity of each approach to distinguish both distantly and closely related strains was assessed. Large genetic distances for both marker types were evident in the phylogenetic trees and population cluster modelling for isolates sampled in distant geographic locations. Both SNP and microsatellite markers distinctly partitioned the eight globally diverse isolates from Sudan, Ethiopia, Kenya, France, China and Sri Lanka from one another, and clearly separated these from the Indian subcontinent *L. donovani* strains (Fig. 2). Notably, the Sri Lankan strains had an intermediate position between the microsatellite-defined populations In^1,2^NpBdLk and KeSdEtIn^3,4^ when the complete set of 193 strains was analysed, but were assigned to the African group (KeSdEtLk) in the analysis on the 33 strains (Fig. 3).

Further resolution of the intraspecific population structure for strains from Nepal and Bihar was only clearly demonstrated by SNP typing, which allowed the discovery of a novel lineage from the Terai region of Nepal in a relatively homogeneous population according to microsatellite results (Fig. 2). These two strains (BPK026/0cl5 and BPK031/0cl12) were taken from patients with VL and yet were more closely related to the Sri Lankan strain from a case of CL than the isolates of *L. infantum* and African *L. donovani* that caused VL or PKDL. The genetic uniqueness of this lineage was first identified in kDNA (Bhattarai et al., 2010), and was also supported by MLMT here that phylogenetically differentiated this pair along with BPK406/6 from the rest of the Nepal-Bihar cluster (Fig. 1).

The SNP-based genetic distances between the *L. infantum* and African *L. donovani* used here as outgroup strains were generally greater than those within the cohort from Nepal and India. Although our set of SNPs was originally designed for elucidating diversity among *L. donovani* from the Indian subcontinent, it also resolved the phylogenetic relationships of other strains of the *L. donovani* complex. However, the relative genetic distances between the outgroup strains and Nepalese-Indian population as measured by microsatellites and SNPs differed. Thus, microsatellites remain an effective means of identifying major genetic groups in a quantifiable manner, though clearly our panel of SNPs was superior in resolving strains within geographical populations.

In contrast to the limited resolving capacity of microsatellites within the Indian subcontinent population, this study presented clear evidence of the power of SNPs to discriminate between strains in a phenotypically diverse population of *L. donovani*. Moreover, certain strains presented high numbers of unique SNPs (like BPK067/0cl2 and BPK294/0cl1) suggesting the presence of further distinct clusters of diversity and highlighting a need for deeper sampling not apparent from microsatellite analysis alone. Furthermore, strains from Calcutta in India (LRC-L51a, LRC-L51p, SC23) clustered with the Kenyan ones and were genetically distinct from other Indian subcontinent strains, underscoring the potential for divergent lineages from the same endemic focus to be infectious in addition to illustrating the need to probe further the ancestry of Indian and African *Leishmania*.

### 3.4 Functional variation in the *L. donovani* complex

An advantage of using SNPs to characterise evolutionary and population variation is the inclusion of protein-level sequence substitutions that can elicit functionally relevant changes (Supplementary Fig. 5). Here, we examined mutations differing homozygously between groups with significant protein impact prediction confidence. Three SNPs discriminating between populations were predicted to significantly affect protein tertiary structure (Table 2). The first of these was a P860L change that was unique to populations In/Np 1, 2 and 3 along with strain 597LN in the 5′ A2-related gene (LdBPK_220660), upstream of the A2 locus. This gene is expressed only during host infection (Ghedin et al., 1998; Zhang and Matlashewski, 2001), is involved in promastigote-amastigote stage differentiation (Alcolea et al., 2010), and here also had a heterozygous substitution (T1036M) partitioning the same groups with a significant predicted functional impact. Two other amino acid mutations in mannosyltransferase (LdBPK_311920) and fatty acid desaturase (LdBPK_261680) genes also differentiated the In/Np 1, 2 and 3 set from the remainder.

Three protein-level mutations observed here occurred in genes previously implicated in antimonial resistance, though only one could distinguish to some extent the *in vitro* SSG phenotypes. This mutation in the rhomboid protein serine peptidase gene (LdBPK_040850) was predicted to have a significant functional impact (A113V) and separated a distinct population composed solely of SSG-resistant strains (In/Np 3) from the others. Furthermore, none of the patients from which the In/Np 3 strains were isolated responded to SSG treatment. The first of the two other variants was a S890N polymorphism unique to population In/Np 2 in a gene encoding ubiquitin-activating enzyme e1 (LdBPK_230710) that has been linked with resistance *in vitro* (Salotra et al., 2006). This gene product was more highly expressed in bone marrow aspirates of VL patients than in skin lesions of either PKDL or CL patients, implying a possible involvement in disease pathology (Sharma et al., 2010). The second change (G130S) was unique to BPK294/0cl1 and occurred in a tryparedoxin gene (LdBPK_291220) whose product is immunogenic in hosts (Cabral et al., 2008) and is more highly expressed in strains resistant to antimonials (Wyllie et al., 2010). The protein is associated with defence against oxidative stress, like that from anti-leishmanial drugs, by maintaining thiol metabolism, and the associated tryparedoxin pathway may be subject to directional selection (Downing et al., 2011).

The phylogenetic relationship revealed by genome-wide SNP typing differed slightly from previous kDNA work (Bhattarai et al., 2010): In/Np 2, along with BPK035/0cl1 and BPK043/0cl2 from In/Np 1, formed a separate cluster from In/Np 1 and 3 (Supplementary Fig. 7). Considering the differing modes of inheritance between kDNA and chromosomal DNA this might reflect recombination among closely related strains. The low level of admixture in the strains BPK035/0cl1 and BPK043/0cl2 for the clusters In/Np 1 and 2 (Fig. 3) suggests that a wider panel of SNPs may be required to test this. Additionally, these two strains could represent another functionally and genetically distinct focus of *L. donovani* diversity from this area (Supplementary Fig. 4) given their high number of unique alleles (such as G443E in protein kinase gene LdBPK_271290).

A protein-level mutation (V185M) in the NADH-flavin oxidoreductase/NADH oxidase gene (LdBPK_120730) unique to BPK026/0cl5, BPK031/0cl12 and the two Kenyan strains (NLB218 and LRC-L53) may be involved in promastigote-amastigote development (Rosenzweig et al., 2008). The V185 variant was verified in Illumina reads for BPK026/0cl5 mapped to the reference *L. donovani* genome sequence (unpublished data); in contrast, the Spanish reference *L. infantum* genome for JPCM5 has the M185 allele (http://www.genedb.org). Finally, a G443E substitution in a gene encoding a protein kinase (LdBPK_271290) unique to BPK035/0cl1 and BPK043/0cl2 had a significant predicted functional impact.

## 4 Conclusions

We investigated the genetic relationships of *Leishmania* using traditional microsatellite and genome-wide SNP markers in order to resolve variation in strains from Nepal and India and the wider *L. donovani* species complex, and to compare the power of these two marker systems. In this study, we found that microsatellites present strains from the Bihar state of India and Terai region of Nepal as a single homogeneous cluster, like previous studies using MLMT (Alam et al., 2009). In contrast, SNP data provided compelling evidence that multiple lineages are circulating in this small region. It is clear that more genetically divergent and novel lineages will be observed in regions endemic for VL with more comprehensive sampling.

The hypothesis of a population bottleneck followed by a recent clonal expansion of strains from Terai and Bihar is supported here by the nodular and star-like structure of the SNP-based phylogenetic networks. Furthermore, simulations of the population history were symptomatic of such a scenario, and intriguingly the peak bottleneck point falls soon after the insecticide-driven parasite elimination programs in the 1960s. However, our survey here indicates that neither the number of expansions in this small geographic area nor the number of origins of drug resistance can be clearly resolved without deeper and wider sampling. The proposed recent population contraction needs to be considered in a framework of likely cyclical population changes associated with drug-driven directional selection for new advantageous alleles, which may be accelerated by the increased fitness in drug-resistant variants from the same area (Vanaerschot et al., 2010; Ouakad et al., 2011). Sharp selective sweeps driven by chemotherapeutic drugs would lead to reduced genetic variation (Kaplan et al., 1989), but here we show evidence of recombination and a distinct hub of diversity in this region, countering the pervasive pattern of genetic homogeneity and lending support to the idea of periodised epidemiological shifts interspersing clonal outbreaks.

Insights on additional factors modulating the evolution of these strains comes from the phylogenetic grouping of strains isolated prior to and following the 1960s parasite elimination schemes in the Indian subcontinent (Fig. 1). Indian strain L13 (from 1961) clustered with Sudan samples from the 1990s, and older Indian isolates (LRC-L51p and SC23, both 1954) grouped with both Kenyan strains of the same era (LRC-L53, 1955; LRC-L57, 1962) and also with more recent ones from India (LRC-L51a, 1971) and Kenya (NLB189, 1983; NKB218, 1984; NLB323, 1985). Coupling these observations with the detection of a divergent lineage from Nepal in this study suggests that the diverse lineages of *L. donovani* that contributed to re-emergence of the leishmaniases as a major disease burden in the Indian subcontinent are likely to persist, and may provide a reservoir of diversity for future epidemics and treatment resistance. Consequently, wider sampling of *Leishmania* parasites resistant to treatment drugs and isolated from asymptomatic infections (Rijal et al., 2010) is required to evaluate this hypothesis.

While the advantages of using genome-wide SNPs as a basis for evaluating population-level variation in *Leishmania* populations are illustrated here, there are limitations to SNP-based analyses that require noting. Phylogenetic distances may be skewed by both the under-representation of rare alleles (Clark et al., 2005) and also by adaptive processes (Rosenberg et al., 2003). Additionally, there is an ascertainment bias for quantifying relationships for markers designed to be informative for different datasets; here, we designed a SNP panel to resolve population-level relationships, and consequently the inter-population distances were qualitative rather than quantitative due to the fixation of population-level variants in the outgroup strains (Albrechtsen et al., 2010). Combining the relative power of each marker system by resolving inter-population groups with microsatellites, and intra-population structure using SNPs may help maximise the information output (Hamblin et al., 2007). However, the resolving power will depend on the extent of SNP and microsatellite concordance (Van Inghelandt et al., 2010), as well as the effects of adaptive evolution or population size change (Toro and Caballero, 2005) and the level of genetic differentiation (Rosenberg et al., 2003).

Given the increased power of SNPs compared to microsatellites in clarifying the structure of closely related populations in other species (Morin et al., 2004; Tokarska et al., 2009), this paper highlights the relevance of harnessing new sequencing technologies in exploring the epidemiology of infectious diseases. Moreover, it provides evidence that only complete genomes will provide a comprehensive phylogenetic profile of diversity within and between *Leishmania* species and populations (Van der Auwera et al., 2011), especially in light of the extensive structural variation in *Leishmania* (Downing et al., 2011; Rogers et al., 2011).

## Author contributions

G.S., J.C.D., M.B., O.S. and T.D. designed the study. I.M., M.S., M.V., S.D.D., S.D., S.R. and S.S. collected, documented and maintained samples. H.I., O.S. and T.D. examined sequence data. O.S. and T.D. performed analyses. G.S., J.C.D., M.B., O.S. and T.D. wrote the manuscript.

## Figures and Tables

**Table 1:** Geographic origins, phylogenetic clustering and *in vitro* phenotypes of the strains assessed using both microsatellite and SNP markers.

WHO strain code	*M*/sat^b^	SNP^c^	*M*/sat *K* = 3^d^	Country	SSG^e^
MHOM/CN/1978/D2	6	Inf/Afr/Div	*L. infantum*	China	–
MHOM/ET/1972/GEBRE1	3f	Inf/Afr/Div	KeSdEtIn^3,4^	Ethiopia	–
MHOM/SD/1993/597LN	3g	Inf/Afr/Div	KeSdEtIn^3,4^	Sudan	–
MHOM/FR/1962/LRC-L47	6	Inf/Afr/Div	*L. infantum*	France	–
MHOM/FR/1995/LPN114	6	Inf/Afr/Div	*L. infantum*	France	–
MHOM/KE/1955/LRC-L53	2d	Inf/Afr/Div	KeSdEtIn^3,4^	Kenya	–
MHOM/KE/1984/NLB218	2b	Inf/Afr/Div	KeSdEtIn^3,4^	Kenya	–
MHOM/LK/2002/L60b	4b	Inf/Afr/Div	In^1,2^NpBdLk	Sri Lanka	–
MHOM/IN/2009/BHU568/0cl1^a^	1a	In/Np 3	In^1,2^NpBdLk	India	R
MHOM/IN/2009/BHU573/0cl3^a^	1a	In/Np 3	In1^,2^NpBdLk	India	R
MHOM/NP/2002/BPK025/0cl1	1a	In/Np 1	In^1,2^NpBdLk	Nepal	S
MHOM/NP/2002/BPK026/0cl5	1x	Inf/Afr/Div	In^1,2^NpBdLk	Nepal	S
MHOM/NP/2002/BPK029/0cl6	1a	In/Np 1	In^1,2^NpBdLk	Nepal	S
MHOM/NP/2002/BPK031/0cl12	1z	Inf/Afr/Div	In^1,2^NpBdLk	Nepal	S
MHOM/NP/2002/BPK035/0cl1^a^	1b	In/Np 1	In^1,2^NpBdLk	Nepal	S
MHOM/NP/2002/BPK043/0cl2^a^	1b	In/Np 1	In^1,2^NpBdLk	Nepal	S
MHOM/NP/2002/BPK067/0cl2^a^	1j	In/Np 3	In^1,2^NpBdLk	Nepal	S
MHOM/NP/2002/BPK077/0cl5	1a	In/Np 2	In^1,2^NpBdLk	Nepal	–
MHOM/NP/2002/BPK080/0cl1^a^	1a	In/Np 1	In^1,2^NpBdLk	Nepal	S
MHOM/NP/2002/BPK081/0cl8	1a	In/Np 1	In^1,2^NpBdLk	Nepal	S
MHOM/NP/2002/BPK085/0^a^	1a	In/Np 1	In^1,2^NpBdLk	Nepal	R
MHOM/NP/2002/BPK087/0cl11^a^	1a	In/Np 1	In^1,2^NpBdLk	Nepal	R
MHOM/NP/2002/BPK164/1cl11	1a	In/Np 3	In^1,2^NpBdLk	Nepal	R
MHOM/NP/2002/BPK173/0cl3^a^	1a	In/Np 3	In^1,2^NpBdLk	Nepal	R
MHOM/NP/2002/BPK173/1cl9	1a	In/Np 3	In^1,2^NpBdLk	Nepal	R
MHOM/NP/2002/BPK178/0cl3^a^	1a	In/Np 2	In^1,2^NpBdLk	Nepal	S
MHOM/NP/2003/BPK190/0cl3^a^	1a	In/Np 1	In^1,2^NpBdLk	Nepal	R
MHOM/NP/2003/BPK206/0cl10	1a	In/Np 1	In^1,2^NpBdLk	Nepal	S
MHOM/NP/2003/BPK275/0cl18	1a	In/Np 3	In^1,2^NpBdLk	Nepal	R
MHOM/NP/2003/BPK282/0cl4^a^	1a	In/Np 2	In^1,2^NpBdLk	Nepal	S
MHOM/NP/2003/BPK288/0	1a/1 k	In/Np 2	In^1,2^NpBdLk	Nepal	R
MHOM/NP/2003/BPK294/0cl1^a^	1d	In/Np 2	In^1,2^NpBdLk	Nepal	S
MHOM/NP/2003/BPK298/0cl8^a^	1a	In/Np 1	In^1,2^NpBdLk	Nepal	S

The strains are listed alphabetically according to their WHO strain codes. See Supplementary Table 2 for a complete list of all 193 strains examined.

a Strains with published genome sequences: BPK282/0cl4 is the reference genome strain.

b The microsatellite-determined genotypes: those for BPK026/0cl5 (1x) and BPK031/0cl12 (1z) are new.

c Groups assigned for SNPs for *K* = 3 populations for strains as shown in Fig. 2B: Inf/Afr/Div stands for Kenyan, Sudanese, Ethiopian, Sri Lankan, Chinese and French *L. donovani* complex strains and two Nepalese samples (BPK026/0cl5 and BPK031/0cl12); and In/Np stands for India and Nepal.

d Groups assigned according to microsatellite variability for *K* = 3 populations for 193 samples (Fig. 1): KeSdEtIn^3,4^ stands for Kenyan, Sudanese, Ethiopian and Indian isolates; In^1,2^NpBdLk for Indian, Nepalese, Bangladeshi and Sri Lankan strains.

e *In vitro* phenotype for SSG: resistant (R) or sensitive (S). All samples caused VL except for NLB218 and 597LN (PKDL) and L60b (CL).

**Table 2:** Protein-coding SNPs in known genes differentiating *L. donovani* phylogenetic groups.

Chr	Genome position	Amino acid	Gene product	Gene ID	Differentiated groups
31	930,958	E679G	Mannosyltransferase	LdBPK_311920	In/Np 1, 2, 3; 597LN
22	303,316	P860L^a^	5′ a2rel-related protein^b^	LdBPK_220660	In/Np 1, 2, 3
26	610,539	D14E	Fatty acid/sphingolipid δ-4 desaturase	LdBPK_261680	In/Np 1, 2, 3 (except BPK067/0cl2)
28	1023,334	N839S	Splicing factor 3B subunit 1	LdBPK_282780	In/Np 1 (except BPK025/0cl1)
23	255,065	S890N	Ubiquitin-activating enzyme e1^c^	LdBPK_230710	In/Np 2
29	545,079	S18N	Serine/threonine-protein kinase	LdBPK_291420	In/Np 2
4	337,729	A113V^a^	Rhomboid protein serine peptidase Clan S, family S54	LdBPK_040850	In/Np 3^d^
12	483,640	V185M	NADH: flavin oxido-reductase/NADH oxidase^e^	LdBPK_120730	Kenyan; BPK026/0cl5; BPK031/0cl12
27	509,391	G443E^a^	Protein kinase	LdBPK_271290	BPK035/0cl1; BPK043/0cl2
29	457,404	G130S	Tryparedoxin^f^	LdBPK_291220	BPK294/0cl8
18	233,653	C34Y	RNA binding protein	LdBPK_180590	BPK067/0cl1

Phylogenetically informative protein sequence homozygous SNPs observed in genes with known or proposed functions. The Nepalese clusters were the major groups distinguished by Bayesian clustering (Fig. 3).

a Mutation is predicted to have a significant impact on protein function.

b Involved in promastigote–amastigote stage differentiation (Alcolea et al., 2010).

c ssociated with SSG resistance (Salotra et al., 2006) and disease pathology (Sharma et al., 2010).

d All strains were SSG-resistant.

e Implicated in promastigote-amastigote development (Rosenzweig et al., 2008; Sharma et al., 2010); the Kenyan strains are NLB218 and LRC-L53.

f Associated with antimonial drug resistance (Wyllie et al., 2010) and host immunogenicity (Castro et al., 2004).
