# Supplementary material for: Genome-wide SNP and microsatellite variation illuminate population-level epidemiology in the Leishmania donovani species complex
Source: Infect Genet Evol. 2012 Jan;12(1):149–59. doi: 10.1016/j.meegid.2011.11.005 (PMC3315668; doi:10.1016/j.meegid.2011.11.005)
Supplement: Supplementary data [file mmc1.doc]

**Supplementary Data for “Genome-wide SNP and microsatellite variation unravel population-level *Leishmania donovani* species complex epidemiology”**

**Supplementary Methods**

**Sample collection and DNA isolation**

Species identity of the 33 Nepalese and Indian clones assessed for SNP variation was confirmed by PCR-RFLP analysis of cysteine proteinase b genes (Quispe-Tintaya et al. 2004). The parasites were cloned by the micro-drop method (Van Meirvenne et al. 1975) with the exception of two isolates from Nepal (BPK085/0 and BPK288/0). One sample was a sub-clone of the reference genome strain (BPK282/0cl4) that only differed in the number of *in vitro* passages (90 in addition to 47 for the original clone).

DNA for SNP analysis was previously isolated for 17 *L. donovani* strains (Downing and Imamura et al. 2011) and for the samples from Sri Lanka, Sudan, Ethiopia, Kenya and all three *L. infantum*. For the remaining 13 Nepalese *L. donovani* lines, this same DNA isolation process was repeated as outlined. For microsatellite analysis, parasites were cultured as specified previously (Kuhls et al. 2007, Hubel et al. 2007) and DNA eluted for the 41 new and 29 re-typed samples using a proteinase K and phenol-chloroform approach (Schönian et al. 1996).

***In vitro* drug phenotype assays**

*In vitro* antimonial testing of the 13 *L. donovani* assessed for SNP diversity not already tested in Downing and Imamura et al. (2011) was completed as described previously by Rijal et al. (2007). *In vitro* SSG sensitivity for each line was expressed as an activity index, defined as the ratio of a sample’s ED50 versus that for the SSG-sensitive genome-sequenced reference strain (BPK282/0cl4); see Downing and Imamura et al. (2011) for details. Isolates with an activity index ≤ 2 were sensitive to the drug, and those with an activity index ≥ 3 or higher were classed as resistant to SSG.

**Heterozygous sites ascertainment and aneuploidy in *Leishmania***

One possible limitation of both microsatellite and SNP analyses is the observation of changes in chromosome copy number previously observed in 17 strains examined here (Downing and Imamura et al. 2011). This could conceptually affect the ascertainment of heterozygous alleles for SNPs on chromosomes known to vary in copy number. However, the published dataset of 3,549 genomic SNPs used here was originally verified using conservative SNP calling criteria and by PCR amplification, and no triallelic sites were observed in this data, suggesting that any bias related to aneuploidy is minor for the analyses performed in this study. Additionally, the microsatellites examined here have been established as reliable markers for assessing *Leishmania* variation (Ochsenreither et al. 2006, Kuhls et al. 2007, Alam et al. 2009) and so any errors in interpreting heterozygous loci are expected to be inconsequential.

**Coalescent simulations of ancestral population size**

We simulated the ancestral population size using Beast v1.6.2 (Drummond and Rambaut 2007) for Bayesian skyline plots (Drummond et al. 2005) with 10 steps for 10^7^ MCMC iterations after a burn-in of 10^6^. A HKY+Γ+I substitution model with four Γ categories and a strict clock were applied. MCMC convergence was visually inspected of the posterior values. The effective sample size of each parameter was greater than 100.

**Supplementary Results**

**Genetic changes between clones during passaging or associated with drug resistance**

Variability in number of passages within the population of 25 Nepal and Indian samples accounted for little of the total genetic variation (0.6%). The clustering of the post-treatment BPK035/0cl1 and BPK077/0cl5 with the pre-treatment BPK043/0cl2 and BPK085/0, respectively, suggested no substantial differences; likewise, no SNPs separated the three samples from patient BPK288 (Supplementary Figure 3). A polymorphism observed between clones BPK275/0cl18 and BPK275/0cl3 using SNP genotyping (a single noncoding C/T polymorphism on chr31) was not supported in genome sequence data for BPK275/0cl18 (Downing and Imamura et al. 2011).

**Protein-level mutations in strain sets**

While CL due to *L. donovani* has only been reported from a focus in the western Himalayas in northern India (Sharma et al. 2005), it is more prevalent in Sri Lanka (Karunaweera et al. 2003). As a result, two protein-level mutations unique to the Sri Lankan strain (L60b) in hypothetical genes LdBPK_150870 (D145N) and LdBPK_081130 (R388C) could be related to its pathology or genetic drift due its distance from other samples.

There was little variation in the panel of 130 sites between the pre- and post-treatment clones taken from patient BPK173. Though pre-treatment BPK173/0cl3 and post-treatment BPK173/1cl9 may represent entirely different strains, as evidenced by kDNA (Laurent et al. 2007), the latter was isolated after the patient did not respond to drug SSG treatment and so two synonymous heterozygous SNPs observed here could be relevant to the outcome of antimonial drug treatment. The first of these variants (25H) occurred in a folate-biopterin transporter gene (FBT, LdBPK_355160): a leishmaniasis treatment drug (methotrexate) accesses *Leishmania* cells using the same protein receptors as folate (Kaur et al. 1988), and structural variation at such transport genes has been implicated with methotrexate-resistance in *L. tarentolae* (Kundig et al. 1999, Ouameur et al. 2008). The second mutation occurred at amino acid 1187S in hypothetical gene LdBPK_200480; this was also observed as a heterozygous SNP between isolate BPK085/0 and clones from the same line (BPK085/0cl3 and BPK085/0cl8), suggesting that this might represent either evidence of adaptation to *in vitro* environments or a relaxation of selective constraint.

**Phylogenetic inferences of strains based on SNP, microsatellite and kDNA profiles**

As a result of the combining two marker systems to investigate the population structure of strains from this species complex, it is possible to predict to which SNP-defined population strains that have been assayed for microsatellite or kDNA variation might belong. In this study, BPK067/0cl2 was a distinct outlier and thus may represent an additional VL focus – BPK191/0cl9 is closely related (Bhattarai et al. 2010). BPK192/0, BPK208/0, BPK274/0, BPK276/0, BPK278/0, BPK279/0, BPK280/0 and BPK293/0 may belong to In/Np 1 (Bhattari et al. 2010). BPK181/0 and BPK295/0 are likely to group with In/Np 2; and BPK090/0 and BPK091/0 with In/Np 3, which was composed only of strains resistant to *in vitro* SSG treatment (Bhattari et al. 2010).

**Additional references not in main text**

Belkhir K, Borsa P, Chikhi L, Raufaste N, Catch F. 2002. GENETIX 4.05, software under Windows TM for the genetics of the populations. Laboratory Genome, Populations, Interactions, CNRS UMR 5000, University of Montpellier II, Montpellier (France).

Hubel A, Krobitsch S, Horauf A, Clos J (1997) *Leishmania major* Hsp100 is required chiefly in the mammalian stage of the parasite. Mol. Cell. Biol. 17:5987-5995.

Karunaweera ND, Pratlong F, Siriwardane HV, Ihalamulla RL, Dedet JP. 2003. Sri

Lankan cutaneous leishmaniasis is caused by *Leishmania donovani* zymodeme MON-37. Trans R Soc Trop Med Hyg. 97(4):380-1.

Kaur K, Coons T, Emmett K, Ullman B. Methotrexate-resistant *Leishmania donovani* genetically deficient in the folate-methotrexate transporter. J Biol Chem. 1988 263(15):7020-8.

Kündig C, Haimeur A, Légaré D, Papadopoulou B, Ouellette M. Increased transport of pteridines compensates for mutations in the high affinity folate transporter and contributes to methotrexate resistance in the protozoan parasite *Leishmania* *tarentolae*. EMBO J. 1999 18(9):2342-51.

Ouameur AA, Girard I, Légaré D, Ouellette M. Functional analysis and complex gene rearrangements of the folate/biopterin transporter (FBT) gene family in the protozoan parasite *Leishmania*. Mol Biochem Parasitol. 2008 162(2):155-64.

Quispe Tintaya KW, Ying X, Dedet JP, Rijal S, De Bolle X, Dujardin JC. 2004. Antigen genes for molecular epidemiology of leishmaniasis: polymorphism of cysteine proteinase B and surface metalloprotease glycoprotein 63 in the *Leishmania donovani* complex. *J Infect Dis*. 189(6):1035-43.

Rijal S, Yardley V, Chappuis F, Khanal B, Singh R, Boelaert M, De Doncker S, Croft S, Decuypere S, Dujardin JC. 2007. Antimonial treatment of visceral leishmaniasis: are current in vitro susceptibility assays adequate for prognosis of in vivo therapy outcome? *Microbes and Infection*. 9(4):529-35.

Schönian G, Schweynoch C, Zlateva K, Oskam L, Kroon N, Gräser Y, Presber W (1996) Identification and determination of the relationships of species and strains within the genus *Leishmania* using single primers in the polymerase chain reaction. Mol Biochem Parasitol. 77(1):19-29.

Sharma NL, Mahajan VK, Kanga A, Sood A, Katoch,VM, Mauricio I, Singh CD, Parwan UC, Sharma VK, Sharma RC. 2005. Localized cutaneous leishmaniasis due to *Leishmania donovani* and *Leishmania tropica*: preliminary findings of the study of 161 new cases from a new endemic focus in himachal pradesh, India. Am. J. Trop. Med. Hyg. 72:819-824.

Van Meirvenne N, Janssens PG, Magnus E. 1975. Antigenic variation in syringe passaged populations of *Trypanosoma* (Trypanozoon) *brucei.* 1. Rationalization of the experimental approach. *Ann Soc Belg Med Trop*. 55(1):1-23.

**Tables**

Supplementary Table 1. A list of the 15 microsatellite markers used to characterise 41 newly typed and 29 re-typed strains from the *L. donovani* species complex.

| Microsatellite marker | Repeat | Repeat number | Fragment size (bp) | Chr | A | He | Ho |
| --- | --- | --- | --- | --- | --- | --- | --- |
| Lm2TG | TG | 9-28 | 110-148 | 1 | 10 | 0.19 | 0.01 |
| TubCA | CA | 8-17 | 78-96 | 34 | 9 | 0.31 | 0.01 |
| Lm4TA | TA | 6-16 | 67-87 | 1 | 11 | 0.34 | 0.03 |
| B_(Li7-17) | CA | 7-17 | 84-104 | 36 | 10 | 0.30 | 0.04 |
| C_(Li36-67) | CA | 6-9 | 74-80 | 31 | 4 | 0.13 | 0.02 |
| E_(Li22-35) | CA | 5-28 | 78-124 | 1 | 12 | 0.47 | 0.02 |
| F_(Li23-41) | GT | 6-32 | 65-117 | 25 | 23 | 0.44 | 0.08 |
| G_(Li45-24) | CA | 7-20 | 89-115 | 17 | 10 | 0.29 | 0.02 |
| P_(Li71-33) | TG | 6-27 | 95-137 | 31 | 11 | 0.29 | 0.03 |
| Q_(Li71-05) | CA | 7-10 | 106-112 | 35 | 3 | 0.09 | 0.03 |
| R_(Li71-07) | CA | 8-22 | 90-118 | 30 | 7 | 0.26 | 0.01 |
| CS19 | AC | 13-26 | 94-120 | 30 | 8 | 0.28 | 0.01 |
| CS20 | TG | 17-32 | 81-111 | 19 | 12 | 0.27 | 0.04 |
| L7031 | CA | 10-12 | 109-113 | 10 | 9 | 0.19 | 0.03 |
| L7039 | CA | 14-20 | 205-217 | 30 | 9 | 0.29 | 0.01 |

These markers were previously used to type *Leishmania* species (Ochsenreither et al. 2006, Kuhls et al. 2007). Shown here are microsatellite loci with their repeat arrays, fragment size in base pairs and chromosomal location; A (alleles per locus); He (expected heterozygosity); and Ho (observed heterozygosity).

Supplementary Table 2. A list of all strains and their phylogenetic groups.

| WHO code | M/sat ^2^ | SNP ^3^ | M ^5^ | Msat K=3 ^5^ | Country | New ^6^ | Path ^7^ |
| --- | --- | --- | --- | --- | --- | --- | --- |
| MHOM/BD/----/IEDCR1 | 1a |  | 15 | In^1,2^NpBdLk | Bangladesh | R; Alam et al. 2009 | VL |
| MHOM/BD/2006/BD09 | 1a |  | 15 | In^1,2^NpBdLk | Bangladesh | R; Alam et al. 2009 | VL |
| MHOM/BD/2006/BD10 | 1a |  | 15 | In^1,2^NpBdLk | Bangladesh | Alam et al. 2009 | VL |
| MHOM/BD/2006/BD11 | 1a |  | 15 | In^1,2^NpBdLk | Bangladesh | Alam et al. 2009 | PKDL |
| MHOM/BD/2006/BD12 | 1a |  | 15 | In^1,2^NpBdLk | Bangladesh | R; Alam et al. 2009 | VL |
| MHOM/BD/2006/BD14 | 1a |  | 15 | In^1,2^NpBdLk | Bangladesh | R; Alam et al. 2009 | VL |
| MHOM/BD/2006/BD15 | 1a |  | 15 | In^1,2^NpBdLk | Bangladesh | R; Alam et al. 2009 | VL |
| MHOM/BD/2006/BD16 | 1a |  | 15 | In^1,2^NpBdLk | Bangladesh | Alam et al. 2009 | VL |
| MHOM/BD/2006/BD17 | 1a |  | 15 | In^1,2^NpBdLk | Bangladesh | R; Alam et al. 2009 | PKDL |
| MHOM/BD/2006/BD18 | 1f |  | 15 | In^1,2^NpBdLk | Bangladesh | Alam et al. 2009 | VL |
| MHOM/BD/2006/BD19 | 1a |  | 15 | In^1,2^NpBdLk | Bangladesh | Alam et al. 2009 | VL |
| MHOM/BD/2006/BD20 | 1a |  | 15 | In^1,2^NpBdLk | Bangladesh | Alam et al. 2009 | VL |
| MHOM/BD/2006/BD21 | 1a |  | 15 | In^1,2^NpBdLk | Bangladesh | R; Alam et al. 2009 | VL |
| MHOM/BD/2006/BD22 | 1a |  | 15 | In^1,2^NpBdLk | Bangladesh | R; Alam et al. 2009 | VL |
| MHOM/BD/2006/BD23 | 1a |  | 15 | In^1,2^NpBdLk | Bangladesh | Alam et al. 2009 | VL |
| MHOM/BD/2006/BD24 | 1q |  | 15 | In^1,2^NpBdLk | Bangladesh | R; Alam et al. 2009 | VL |
| MHOM/BD/2006/BD25 | 1a |  | 15 | In^1,2^NpBdLk | Bangladesh | R; Alam et al. 2009 | VL |
| MHOM/BD/2006/BD26 | 1a |  | 15 | In^1,2^NpBdLk | Bangladesh | Alam et al. 2009 | VL |
| MHOM/BD/2006/BD27 | 1a |  | 15 | In^1,2^NpBdLk | Bangladesh | R; Alam et al. 2009 | VL |
| MHOM/BD/2006/BD28 | 1a |  | 15 | In^1,2^NpBdLk | Bangladesh | Alam et al. 2009 | VL |
| MHOM/BD/1981/BL1 | 1a |  | 15 | In^1,2^NpBdLk | Bangladesh | Alam et al. 2009 | VL |
| MHOM/CN/2000/Wangjie1 | 6 |  | 15 | *L. infantum* | China | Kuhls et al. 2005 | VL |
| MHOM/CN/1954/Peking | 6 |  | 15 | *L. infantum* | China | Kuhls et al. 2005 | VL |
| MHOM/CN/1978/D2 | 6 | Inf/Afr/Div | 15 | *L. infantum* | China | Kuhls et al. 2005 | VL |
| MHOM/ES/1993/PM1 | 6 |  | 15 | *L. infantum* | Spain | Kuhls et al. 2005 | VL |
| MHOM/ET/----/HUSSEN | 3e |  | 15 | KeSdEtIn^3,4^ | Ethiopia | Kuhls et al. 2005 |  |
| MHOM/ET/1967/HU3 | 5a |  | 15 | KeSdEtIn^3,4^ | Ethiopia | Ochsenreither et al. 2006 | VL |
| MHOM/ET/1972/GEBRE1 | 3f | Inf/Afr/Div | 15 | KeSdEtIn^3,4^ | Ethiopia | Kuhls et al. 2005 | VL |
| MHOM/FR/1962/LRC-L47 | 6 | Inf/Afr/Div | 15 | *L. infantum* | France | Kuhls et al. 2005 |  |
| MHOM/FR/1978/LEM75 | 6 |  | 15 | *L. infantum* | France | Kuhls et al. 2005 |  |
| MHOM/FR/1995/LPN114 | 6 | Inf/Afr/Div | 15 | *L. infantum* | France | Kuhls et al. 2005 |  |
| MHOM/IN/----/BHU569 | 1a |  | 5 | In^1,2^NpBdLk | India | New |  |
| MHOM/IN/----/BHU572 | 1a |  | 5 | In^1,2^NpBdLk | India | New |  |
| MHOM/IN/----/BHU575/0cl2 | 1a |  | 5 | In^1,2^NpBdLk | India | New |  |
| MHOM/IN/----/BHU581/0cl2 | 1a |  | 5 | In^1,2^NpBdLk | India | New |  |
| MHOM/IN/----/BHU592/0cl5 | 1a |  | 5 | In^1,2^NpBdLk | India | New |  |
| MHOM/IN/----/DEVI | 1a |  | 15 | In^1,2^NpBdLk | India | Kuhls et al. 2005 | VL |
| MHOM/IN/----/NARESH-RAI | 1a |  | 15 | In^1,2^NpBdLk | India | Alam et al. 2009 |  |
| MHOM/IN/2000/K111 | 1g |  | 10 | In^1,2^NpBdLk | India | Alam et al. 2009 | VL |
| MHOM/IN/2000/K132 | 1g |  | 10 | In^1,2^NpBdLk | India | Alam et al. 2009 | VL |
| MHOM/IN/2000/P69 | 1g |  | 10 | In^1,2^NpBdLk | India | Alam et al. 2009 | PKDL |
| MHOM/IN/2000/P75 | 1g |  | 10 | In^1,2^NpBdLk | India | Alam et al. 2009 | PKDL |
| MHOM/IN/2000/P82 | 1g |  | 10 | In^1,2^NpBdLk | India | Alam et al. 2009 | PKDL |
| MHOM/IN/2000/P85 | 1g |  | 10 | In^1,2^NpBdLk | India | Alam et al. 2009 | PKDL |
| MHOM/IN/2001/BHU20140 | 1g |  | 15 | In^1,2^NpBdLk | India | Kuhls et al. 2005 | VL |
| MHOM/IN/2001/K149 | 1g |  | 10 | In^1,2^NpBdLk | India | Alam et al. 2009 | VL |
| MHOM/IN/2001/K155 | 1g |  | 10 | In^1,2^NpBdLk | India | Alam et al. 2009 | VL |
| MHOM/IN/2001/P94 | 1g |  | 10 | In^1,2^NpBdLk | India | Alam et al. 2009 | PKDL |
| MHOM/IN/2002/BHU1 | 1a |  | 15 | In^1,2^NpBdLk | India | Kuhls et al. 2005 | VL |
| MHOM/IN/2002/BHU11 | 1a |  | 15 | In^1,2^NpBdLk | India | Kuhls et al. 2005 | VL |
| MHOM/IN/2002/BHU12 | 1a |  | 15 | In^1,2^NpBdLk | India | Kuhls et al. 2005 | VL |
| MHOM/IN/2002/BHU13 | 1a |  | 15 | In^1,2^NpBdLk | India | Kuhls et al. 2005 | VL |
| MHOM/IN/2002/BHU15 | 1a |  | 15 | In^1,2^NpBdLk | India | Kuhls et al. 2005 | VL |
| MHOM/IN/2002/BHU17 | 1a |  | 15 | In^1,2^NpBdLk | India | Kuhls et al. 2005 | VL |
| MHOM/IN/2002/BHU2 | 1a |  | 15 | In^1,2^NpBdLk | India | Kuhls et al. 2005 | VL |
| MHOM/IN/2002/BHU20 | 1a/1b |  | 15 | In^1,2^NpBdLk | India | Alam et al. 2009 | VL |
| MHOM/IN/2002/BHU3 | 1a/1c |  | 15 | In^1,2^NpBdLk | India | Alam et al. 2009 | VL |
| MHOM/IN/2002/BHU4 | 1a |  | 15 | In^1,2^NpBdLk | India | Kuhls et al. 2005 | VL |
| MHOM/IN/2002/BHU5 | 1a |  | 15 | In^1,2^NpBdLk | India | Kuhls et al. 2005 | VL |
| MHOM/IN/2002/BHU6 | 1h |  | 15 | In^1,2^NpBdLk | India | Kuhls et al. 2005 | VL |
| MHOM/IN/2002/BHU7 | 1a/1b |  | 15 | In^1,2^NpBdLk | India | Kuhls et al. 2005 | VL |
| MHOM/IN/2002/BHU8 | 1a |  | 15 | In^1,2^NpBdLk | India | Alam et al. 2009 | VL |
| MHOM/IN/2002/BHU9 | 1a/1b |  | 15 | In^1,2^NpBdLk | India | Kuhls et al. 2005 | VL |
| MHOM/IN/2002/K172 | 1a |  | 10 | In^1,2^NpBdLk | India | Kuhls et al. 2005 | VL |
| MHOM/IN/2003/BHU32 | 1a |  | 15 | In^1,2^NpBdLk | India | Alam et al. 2009 | VL |
| MHOM/IN/2003/BHU33 | 1a |  | 15 | In^1,2^NpBdLk | India | Alam et al. 2009 | VL |
| MHOM/IN/2003/BHU37 | 1a |  | 15 | In^1,2^NpBdLk | India | Alam et al. 2009 | VL |
| MHOM/IN/2003/BHU41 | 1m |  | 15 | In^1,2^NpBdLk | India | Alam et al. 2009 | VL |
| MHOM/IN/2003/BHU50 | 1g |  | 15 | In^1,2^NpBdLk | India | Alam et al. 2009 | VL |
| MHOM/IN/2003/BHU52 | 1g |  | 15 | In^1,2^NpBdLk | India | Alam et al. 2009 | VL |
| MHOM/IN/2003/BHU53 | 1g |  | 15 | In^1,2^NpBdLk | India | Alam et al. 2009 | VL |
| MHOM/IN/2003/BHU54 | 1g |  | 15 | In^1,2^NpBdLk | India | Alam et al. 2009 | VL |
| MHOM/IN/2003/BHU55 | 1g |  | 15 | In^1,2^NpBdLk | India | Alam et al. 2009 | VL |
| MHOM/IN/2003/K216 | 1g |  | 10 | In^1,2^NpBdLk | India | Alam et al. 2009 | VL |
| MHOM/IN/2005/K339 | 1g |  | 10 | In^1,2^NpBdLk | India | Alam et al. 2009 | VL |
| MHOM/IN/2005/P137 | 1g |  | 10 | In^1,2^NpBdLk | India | Alam et al. 2009 | PKDL |
| MHOM/IN/2009/BHU1062/4 | 1a/1b |  | 5 | In^1,2^NpBdLk | India | New | VL |
| MHOM/IN/2009/BHU568/0cl1 ^1^ | 1a | In/Np 3 | 5 | In^1,2^NpBdLk | India | New | VL |
| MHOM/IN/2009/BHU573/0cl3 ^1^ | 1a | In/Np 3 | 5 | In^1,2^NpBdLk | India | New | VL |

Supplementary Table 2 continued.

| WHO code | M/sat ^2^ | SNP ^3^ | M ^4^ | Msat K=3 ^5^ | Country | New ^6^ | Path ^7^ |
| --- | --- | --- | --- | --- | --- | --- | --- |
| MHOM/IN/2009/BHU741/1cl1 | 1a |  | 5 | In^1,2^NpBdLk | India | New | VL |
| MHOM/IN/2009/BHU800/1 | 1a |  | 5 | In^1,2^NpBdLk | India | New | VL |
| MHOM/IN/2009/BHU815/1 | 1a |  | 5 | In^1,2^NpBdLk | India | New | VL |
| MHOM/IN/2009/BHU994/1 | 1a/1s |  | 5 | In^1,2^NpBdLk | India | New | VL |
| MHOM/IN/2010/BHU1042/1 | 1a |  | 5 | In^1,2^NpBdLk | India | New | VL |
| MHOM/IN/2010/BHU1080/1 | 1a |  | 5 | In^1,2^NpBdLk | India | New | VL |
| MHOM/IN/2010/BHU1093/1 | 1a |  | 5 | In^1,2^NpBdLk | India | New | VL |
| MHOM/IN/2010/BHU1113/7 | 1a |  | 5 | In^1,2^NpBdLk | India | New | VL |
| MHOM/IN/2010/BHU1121/1 | 1a |  | 5 | In^1,2^NpBdLk | India | New | VL |
| MHOM/IN/2010/BHU764/0cl1 | 1a |  | 5 | In^1,2^NpBdLk | India | New | VL |
| MHOM/IN/2010/BHU770/0cl1 | 1a |  | 5 | In^1,2^NpBdLk | India | New | VL |
| MHOM/IN/2010/BHU777/0cl1 | 1i |  | 5 | In^1,2^NpBdLk | India | New | VL |
| MHOM/IN/2010/BHU782/0cl1 | 1a |  | 5 | In^1,2^NpBdLk | India | New | VL |
| MHOM/IN/2010/BHU796/1 | 1a |  | 5 | In^1,2^NpBdLk | India | New | VL |
| MHOM/IN/2010/BHU814/1 | 1a |  | 5 | In^1,2^NpBdLk | India | New | VL |
| MHOM/IN/2010/BHU872/6 | 1a |  | 5 | In^1,2^NpBdLk | India | New | VL |
| MHOM/IN/2010/BHU902/1 | 1a |  | 5 | In^1,2^NpBdLk | India | New | VL |
| MHOM/IN/1954/LRC-L51p | 2a |  | 15 | KeSdEtIn^3,4^ | India | Kuhls et al. 2005 |  |
| MHOM/IN/1954/SC23 | 2a |  | 15 | KeSdEtIn^3,4^ | India | Kuhls et al. 2005 | VL |
| MHOM/IN/1961/L13 | 3a |  | 15 | KeSdEtIn^3,4^ | India | Kuhls et al. 2005 | PKDL |
| MHOM/IN/1971/LRC-L51a | 2a |  | 15 | KeSdEtIn^3,4^ | India | Kuhls et al. 2005 |  |
| MHOM/IN/1977/Chowdhury-III | 1a/1e |  | 15 | In^1,2^NpBdLk | India | Alam et al. 2009 | VL |
| MHOM/IN/1977/Chowdhury-IV | 1a |  | 15 | In^1,2^NpBdLk | India | Alam et al. 2009 | VL |
| MHOM/IN/1977/Chowdhury-V | 1a |  | 15 | In^1,2^NpBdLk | India | Alam et al. 2009 | VL |
| MHOM/IN/1977/Chowdhury-X | 1a |  | 15 | In^1,2^NpBdLk | India | Alam et al. 2009 | VL |
| MHOM/IN/1977/MUNNI | 1a |  | 15 | In^1,2^NpBdLk | India | Alam et al. 2009 | VL |
| MHOM/IN/1978/STL2-78 | 1a |  | 15 | In^1,2^NpBdLk | India | Alam et al. 2009 | VL |
| MHOM/IN/1979/DD5 | 1i |  | 15 | In^1,2^NpBdLk | India | Alam et al. 2009 | VL |
| MHOM/IN/1979/STL1-79 | 1i |  | 15 | In^1,2^NpBdLk | India | Alam et al. 2009 | VL |
| MHOM/IN/1979/STL2-79 | 1a |  | 15 | In^1,2^NpBdLk | India | Alam et al. 2009 | VL |
| MHOM/IN/1980/DD8 | 1b |  | 15 | In^1,2^NpBdLk | India | R; Ochsenreither et al. 2006 | VL |
| MHOM/IN/1980/STL39-80 | 1a |  | 15 | In^1,2^NpBdLk | India | Alam et al. 2009 | PKDL |
| MHOM/IN/1981/STL106-81 | 1a |  | 15 | In^1,2^NpBdLk | India | Alam et al. 2009 | PKDL |
| MHOM/IN/1982/NANDI1 | 1a |  | 15 | In^1,2^NpBdLk | India | Alam et al. 2009 | VL |
| MHOM/IN/1982/NANDI2 | 1a |  | 15 | In^1,2^NpBdLk | India | Alam et al. 2009 | VL |
| MHOM/IN/1982/NANDI3 | 1a |  | 15 | In^1,2^NpBdLk | India | Alam et al. 2009 | PKDL |
| MHOM/IN/1983/Chandigarh | 4a |  | 15 | In^1,2^NpBdLk | India | Alam et al. 2009 | VL |
| MHOM/IN/1993/B12302 | 1a |  | 15 | In^1,2^NpBdLk | India | Alam et al. 2009 |  |
| MHOM/IN/1996/THAK35 | 1a |  | 15 | In^1,2^NpBdLk | India | Ochsenreither et al. 2006 |  |
| MHOM/IN/1998/P21 | 1g |  | 10 | In^1,2^NpBdLk | India | Alam et al. 2009 | PKDL |
| MHOM/IN/1998/P48 | 1g |  | 10 | In^1,2^NpBdLk | India | Alam et al. 2009 | PKDL |
| MHOM/IN/1999/K59 | 1g |  | 10 | In^1,2^NpBdLk | India | Alam et al. 2009 | VL |
| MHOM/IN/1999/K75 | 1g |  | 10 | In^1,2^NpBdLk | India | Alam et al. 2009 | VL |
| MHOM/IN/1999/K80 | 1g |  | 10 | In^1,2^NpBdLk | India | Alam et al. 2009 | VL |
| MHOM/IN/1999/P49 | 1g |  | 10 | In^1,2^NpBdLk | India | Alam et al. 2009 | PKDL |
| MHOM/KE/----/LRC-L445 | 2e |  | 15 | KeSdEtIn^3,4^ | Ethiopia | Ochsenreither et al. 2006 |  |
| MHOM/KE/1955/LRC-L53 | 2d | Inf/Afr/Div | 15 | KeSdEtIn^3,4^ | Kenya | Ochsenreither et al. 2006 |  |
| MHOM/KE/1962/LRC-L57 | 2g |  | 15 | KeSdEtIn^3,4^ | Kenya | Ochsenreither et al. 2006 |  |
| MHOM/KE/1973/MRC74 | 2f |  | 15 | KeSdEtIn^3,4^ | Kenya | Ochsenreither et al. 2006 |  |
| MHOM/KE/1983/NLB 189 | 2a |  | 15 | KeSdEtIn^3,4^ | Kenya | Ochsenreither et al. 2006 | PKDL |
| MHOM/KE/1984/NLB 218 | 2b | Inf/Afr/Div | 15 | KeSdEtIn^3,4^ | Kenya | Ochsenreither et al. 2006 | PKDL |
| MHOM/KE/1985/NLB 323 | 2c |  | 15 | KeSdEtIn^3,4^ | Kenya | Ochsenreither et al. 2006 | VL |
| MHOM/LK/2002/L60b | 4b | Inf/Afr/Div | 15 | In^1,2^NpBdLk | Sri Lanka | Alam et al. 2009 | CL |
| MHOM/LK/2002/L60c | 4b |  | 15 | In^1,2^NpBdLk | Sri Lanka | Alam et al. 2009 | CL |
| MHOM/NP/2002/BPK025/0cl1 | 1a | In/Np 1 | 15 | In^1,2^NpBdLk | Nepal | Alam et al. 2009 | VL |
| MHOM/NP/2002/BPK026/0cl5 | 1x | Inf/Afr/Div | 5 | In^1,2^NpBdLk | Nepal | R; Bhattarai et al. 2010 | VL |
| MHOM/NP/2002/BPK029/0cl6 | 1a | In/Np 1 | 5 | In^1,2^NpBdLk | Nepal | Bhattarai et al. 2010 | VL |
| MHOM/NP/2002/BPK031/0cl12 | 1z | Inf/Afr/Div | 5 | In^1,2^NpBdLk | Nepal | R; Bhattarai et al. 2010 | VL |
| MHOM/NP/2002/BPK035/0cl1 ^1^ | 1b | In/Np 1 | 15 | In^1,2^NpBdLk | Nepal | Alam et al. 2009 | VL |
| MHOM/NP/2002/BPK043/0cl2 ^1^ | 1b | In/Np 1 | 15 | In^1,2^NpBdLk | Nepal | Alam et al. 2009 | VL |
| MHOM/NP/2002/BPK067/0cl2 ^1^ | 1j | In/Np 3 | 5 | In^1,2^NpBdLk | Nepal | Bhattarai et al. 2010 | VL |
| MHOM/NP/2002/BPK077/0cl5 | 1a | In/Np 2 | 15 | In^1,2^NpBdLk | Nepal | Alam et al. 2009 | VL |
| MHOM/NP/2002/BPK080/0cl1 ^1^ | 1a | In/Np 1 | 15 | In^1,2^NpBdLk | Nepal | Alam et al. 2009 | VL |
| MHOM/NP/2002/BPK081/0cl8 | 1a | In/Np 1 | 5 | In^1,2^NpBdLk | Nepal | New | VL |
| MHOM/NP/2002/BPK085/0 ^1^ | 1a | In/Np 1 | 15 | In^1,2^NpBdLk | Nepal | Alam et al. 2009 | VL |
| MHOM/NP/2002/BPK087/0cl11 ^1^ | 1a | In/Np 1 | 5 | In^1,2^NpBdLk | Nepal | R; Bhattarai et al. 2010 | VL |
| MHOM/NP/2002/BPK090/0cl4 | 1a |  | 15 | In^1,2^NpBdLk | Nepal | Alam et al. 2009 | VL |
| MHOM/NP/2002/BPK091/0cl9 | 1a |  | 15 | In^1,2^NpBdLk | Nepal | Alam et al. 2009 | VL |
| MHOM/NP/2002/BPK157/0cl5 | 1a |  | 5 | In^1,2^NpBdLk | Nepal | New | VL |
| MHOM/NP/2002/BPK158/0cl9 | 1a |  | 5 | In^1,2^NpBdLk | Nepal | New | VL |
| MHOM/NP/2002/BPK164/1cl11 | 1a | In/Np 3 | 15 | In^1,2^NpBdLk | Nepal | Alam et al. 2009 | VL |
| MHOM/NP/2002/BPK173/0cl3 ^1^ | 1a | In/Np 3 | 5 | In^1,2^NpBdLk | Nepal | R; Bhattarai et al. 2010 | VL |
| MHOM/NP/2002/BPK173/1cl9 | 1a | In/Np 3 | 5 | In^1,2^NpBdLk | Nepal | R; Bhattarai et al. 2010 | VL |
| MHOM/NP/2002/BPK177/0cl7 | 1a |  | 15 | In^1,2^NpBdLk | Nepal | R; Alam et al. 2009 | VL |
| MHOM/NP/2002/BPK178/0cl3 ^1^ | 1a | In/Np 2 | 15 | In^1,2^NpBdLk | Nepal | Alam et al. 2009 | VL |
| MHOM/NP/2003/BPK181/0cl1 | 1a |  | 5 | In^1,2^NpBdLk | Nepal | Bhattarai et al. 2010 | VL |
| MHOM/NP/2003/BPK190/0cl3 ^1^ | 1a | In/Np 1 | 15 | In^1,2^NpBdLk | Nepal | R; Alam et al. 2009 | VL |
| MHOM/NP/2003/BPK191 | 1j |  | 15 | In^1,2^NpBdLk | Nepal | Alam et al. 2009 | VL |
| MHOM/NP/2003/BPK192 | 1j |  | 5 | In^1,2^NpBdLk | Nepal | Bhattarai et al. 2010 | VL |
| MHOM/NP/2003/BPK206/0cl10 ^1^ | 1a | In/Np 1 | 15 | In^1,2^NpBdLk | Nepal | R; Alam et al. 2009 | VL |
| MHOM/NP/2003/BPK208/0cl2 | 1a |  | 15 | In^1,2^NpBdLk | Nepal | Alam et al. 2009 | VL |
| MHOM/NP/2003/BPK274/0cl5 | 1a |  | 5 | In^1,2^NpBdLk | Nepal | R; Bhattarai et al. 2010 | VL |
| MHOM/NP/2003/BPK275/0cl18 ^1^ | 1a | In/Np 3 | 15 | In^1,2^NpBdLk | Nepal | R; Alam et al. 2009 | VL |
| MHOM/NP/2003/BPK276/0cl6 | 1a/1b |  | 15 | In^1,2^NpBdLk | Nepal | R; Alam et al. 2009 | VL |

Supplementary Table 2 continued.

| WHO code | M/sat ^2^ | SNP ^3^ | M ^4^ | Msat K=3 ^5^ | Country | New ^6^ | Path ^7^ |
| --- | --- | --- | --- | --- | --- | --- | --- |
| MHOM/NP/2003/BPK278 | 1j |  | 5 | In^1,2^NpBdLk | Nepal | Bhattarai et al. 2010 | VL |
| MHOM/NP/2003/BPK279/0cl6 | 1a |  | 15 | In^1,2^NpBdLk | Nepal | R; Alam et al. 2009 | VL |
| MHOM/NP/2003/BPK280 | 1a |  | 5 | In^1,2^NpBdLk | Nepal | Alam et al. 2009 | VL |
| MHOM/NP/2003/BPK282/0cl4 ^1^ | 1a | In/Np 2 | 15 | In^1,2^NpBdLk | Nepal | R; Alam et al. 2009 | VL |
| MHOM/NP/2003/BPK288/0 | 1a/1k | In/Np 2 | 5 | In^1,2^NpBdLk | Nepal | R; Bhattarai et al. 2010 | VL |
| MHOM/NP/2003/BPK293 | 1a |  | 5 | In^1,2^NpBdLk | Nepal | Bhattarai et al. 2010 | VL |
| MHOM/NP/2003/BPK294/0cl1 ^1^ | 1d | In/Np 2 | 15 | In^1,2^NpBdLk | Nepal | R; Alam et al. 2009 | VL |
| MHOM/NP/2003/BPK295/0cl6 | 1a |  | 5 | In^1,2^NpBdLk | Nepal | R; Bhattarai et al. 2010 | VL |
| MHOM/NP/2003/BPK298/0cl8 ^1^ | 1a | In/Np 1 | 5 | In^1,2^NpBdLk | Nepal | R; Bhattarai et al. 2010 | VL |
| MHOM/NP/2004/NEP021/6 | 1a/1k |  | 5 | In^1,2^NpBdLk | Nepal | New | VL |
| MHOM/NP/2004/NEP052/3 | 1a |  | 5 | In^1,2^NpBdLk | Nepal | New | VL |
| MHOM/NP/2004/NEP098/6 | 1a |  | 5 | In^1,2^NpBdLk | Nepal | New | VL |
| MHOM/NP/2004/NEP107/6 | 1a |  | 5 | In^1,2^NpBdLk | Nepal | New | VL |
| MHOM/NP/2004/NEP123/6 | 1a |  | 5 | In^1,2^NpBdLk | Nepal | New | VL |
| MHOM/NP/2009/BPK455/0 | 1a |  | 5 | In^1,2^NpBdLk | Nepal | New | VL |
| MHOM/NP/2009/BPK506/0 | 1a |  | 5 | In^1,2^NpBdLk | Nepal | New | VL |
| MHOM/NP/2010/BPK406/6 | 1y |  | 5 | In^1,2^NpBdLk | Nepal | New | VL |
| MHOM/NP/2010/BPK455/2 | 1a |  | 5 | In^1,2^NpBdLk | Nepal | New | VL |
| MHOM/NP/2010/BPK512/0 | 1w |  | 5 | In^1,2^NpBdLk | Nepal | New | VL |
| MHOM/NP/2010/BPK513/0 | 1a |  | 5 | In^1,2^NpBdLk | Nepal | New | VL |
| MHOM/NP/2010/BPK514/0 | 1a |  | 5 | In^1,2^NpBdLk | Nepal | New | VL |
| MHOM/NP/2010/BPK524/0 | 1a |  | 5 | In^1,2^NpBdLk | Nepal | New | VL |
| MHOM/SD/1982/GILANI | 5b |  | 15 | In^1,2^NpBdLk | Sudan | Ochsenreither et al. 2006 | VL |
| MHOM/SD/1993/35-band | 5c |  | 15 | KeSdEtIn^3,4^ | Sudan | Kuhls et al. 2005 | VL |
| MHOM/SD/1993/597LN | 3g | Inf/Afr/Div | 15 | KeSdEtIn^3,4^ | Sudan | Kuhls et al. 2007 | PKDL |
| MHOM/SD/1993/9S | 5d |  | 15 | KeSdEtIn^3,4^ | Sudan | Kuhls et al. 2005 | VL |
| MHOM/SD/1993/GE | 3b |  | 15 | KeSdEtIn^3,4^ | Sudan | Kuhls et al. 2005 | VL |
| MHOM/SD/1997/LEM3429 | 3c |  | 15 | KeSdEtIn^3,4^ | Sudan | Ochsenreither et al. 2006 | VL |
| MHOM/SD/1997/LEM3463 | 3d |  | 15 | KeSdEtIn^3,4^ | Sudan | Ochsenreither et al. 2006 |  |
| MHOM/TN/1980/IPT1 | 6 |  | 15 | *L. infantum* | Tunisia | Kuhls et al. 2007 | VL |
| MHOM/NP/2002/BPK085/0cl8 * | 1a | In/Np 2 | - | In^1,2^NpBdLk | Nepal | - | VL |
| MHOM/NP/2003/BPK275/0cl3 * | 1a | In/Np 3 | - | In^1,2^NpBdLk | Nepal | - | VL |
| MHOM/NP/2003/BPK288/0cl7 *^,1,8^ | 1a/1k | In/Np 2 | - | In^1,2^NpBdLk | Nepal | - | VL |
| MHOM/NP/2003/BPK288/0cl9 * | 1a/1k | In/Np 2 | - | In^1,2^NpBdLk | Nepal | - | VL |
| MHOM/NP/2002/BPK085/0cl3 * | 1a | In/Np 2 | - | In^1,2^NpBdLk | Nepal | - | VL |

The 198 samples are listed alphabetically according to their WHO strain codes. All were genotyped for their microsatellite profile except the last five (marked *). ^1^ Strains with published genome sequences where the strain codes refer to the isolate (where present) and the clone (cl, where present). ^2^ The genetic group assigned based on microsatellite variation: a set of new microsatellite genotypes were observed for strains MHOM/IN/2009/BHU994/1 (1a/1s), MHOM/NP/2002/BPK026/0cl5 (1x), MHOM/NP/2002/BPK031/0cl12 (1z) and MHOM/NP/2010/BPK512/0 (1w). ^3^ Groups assigned for SNPs for four major Structure-defined populations for 33 samples as shown in Figure 2: Inf/Afr/Div stands for *L. infantum*, African, Sri Lankan and two Nepalese samples (BPK026/0cl5 and BPK031/0cl12); In/Np 1, 2 and 3 for India and Nepal populations. ^4^ Number of microsatellite markers used (5, 10 or 15). ^5^ Groups based on microsatellite variability for three main Structure-defined populations: In^1,2^NpBdLk (the main Indian cluster, Nepalese, Bangladeshi and Sri Lankan strains, one strain from northern India); KeSdEtIn^3,4^ (Kenya, Sudan, Ethiopia and four Indian stains); and *L. infantum*. ^6^ The samples newly microsatellite typed in study (New) or previously published but re-typed here (R). ^7^ Disease pathology: visceral (VL), cutaneous (CL), post kala-azar dermal leishmaniasis (PKDL). ^8^ SSG phenotype differed *in vitro* from original isolate; SSG-sensitive clone BPK288/0cl7 underwent nine passages after being taken from isolate SSG-resistant BPK288/0 before sequencing: this difference likely reflects genetic variation within the parasites in isolate BPK288/0; clone BPK288/0cl9 was not yet tested for *in vitro* SSG resistance. All other clones match the phenotypes of the mother isolates listed in Table 1.

Supplementary Table 3. Characterisation of populations identified by microsatellite typing for 193 strains of the *L. donovani* complex from different endemic foci.

| K=3 | N | P | MNA | A | He | Ho |
| --- | --- | --- | --- | --- | --- | --- |
| In^1,2^NpBdLk | 161 | 0.6 | 3.467 | 19 | 0.065 | 0.005 |
| KeSdEtIn^3,4^ | 24 | 1.0 | 4.933 | 20 | 0.643 | 0.159 |
| *L. infantum* | 8 | 1.0 | 4.267 | 8 | 0.667 | 0.050 |

Populations (K=3) corresponding to those identified with Structure. N stands for the number of individuals, P for the proportion of polymorphic loci, MNA for the mean number of alleles, A for the number of alleles (including heterozygous ones), He for the expected heterozygosity and Ho for the observed heterozygosity.

Supplementary Table 4. Identifying population cluster for 130 genome-wide SNPs in 33 *L. donovani* complex strains.

| K ^1^ | L(K) ^2^ | Var ^3^ | L(K)' ^4^ | ∆K ^5^ |
| --- | --- | --- | --- | --- |
| 1 | -3699.1 | 65.7 | - | - |
| 2 | -2890.5 | 177 | 808.6 | -512.6 |
| 3 | -2594.5 | 282 | 296 | -331.3 |
| 4 | -2629.8 | 419 | -35.3 | 46.8 |
| 5 | -2618.3 | 403 | 11.5 | 107.2 |
| 6 | -2499.6 | 398.6 | 118.7 | -118.6 |
| 7 | -2499.5 | 397.8 | 0.1 | -1.1 |
| 8 | -2500.5 | 398.3 | -1 | 0.1 |
| 9 | -2501.4 | 398.3 | -0.9 | -0.1 |
| 10 | -2502.4 | 398.9 | -1 | -0.5 |

Population clustering was repeated for 10 iterations and thus the values represent the means of the trials. A burn in of 10^5^ and 2 x 10^6^ MCMC steps were used. ^1^ The simulated number of populations. ^2^ The mean unadjusted likelihood. ^3^ The mean variance of each set of likelihoods. ^4^ The mean change in likelihood values with respect to K. ^4^ The mean relative rate of change of the likelihood values with respect to K (Evanno et al. 2005). The peak ∆K was at K=5. L(K) did not change noticeably for K > 10. Repeating the same analysis with the five duplicate clones in addition to the 33 strains produced identical results with respect to K and clustering probabilities. No strains were assigned to the fifth population for K=5 and so the number of phylogenetically informative groups was four.

**Figures**

Supplementary Figure 1. Geographic distribution of *L. donovani* species complex samples isolated (A) globally and (B) within Nepal and India.


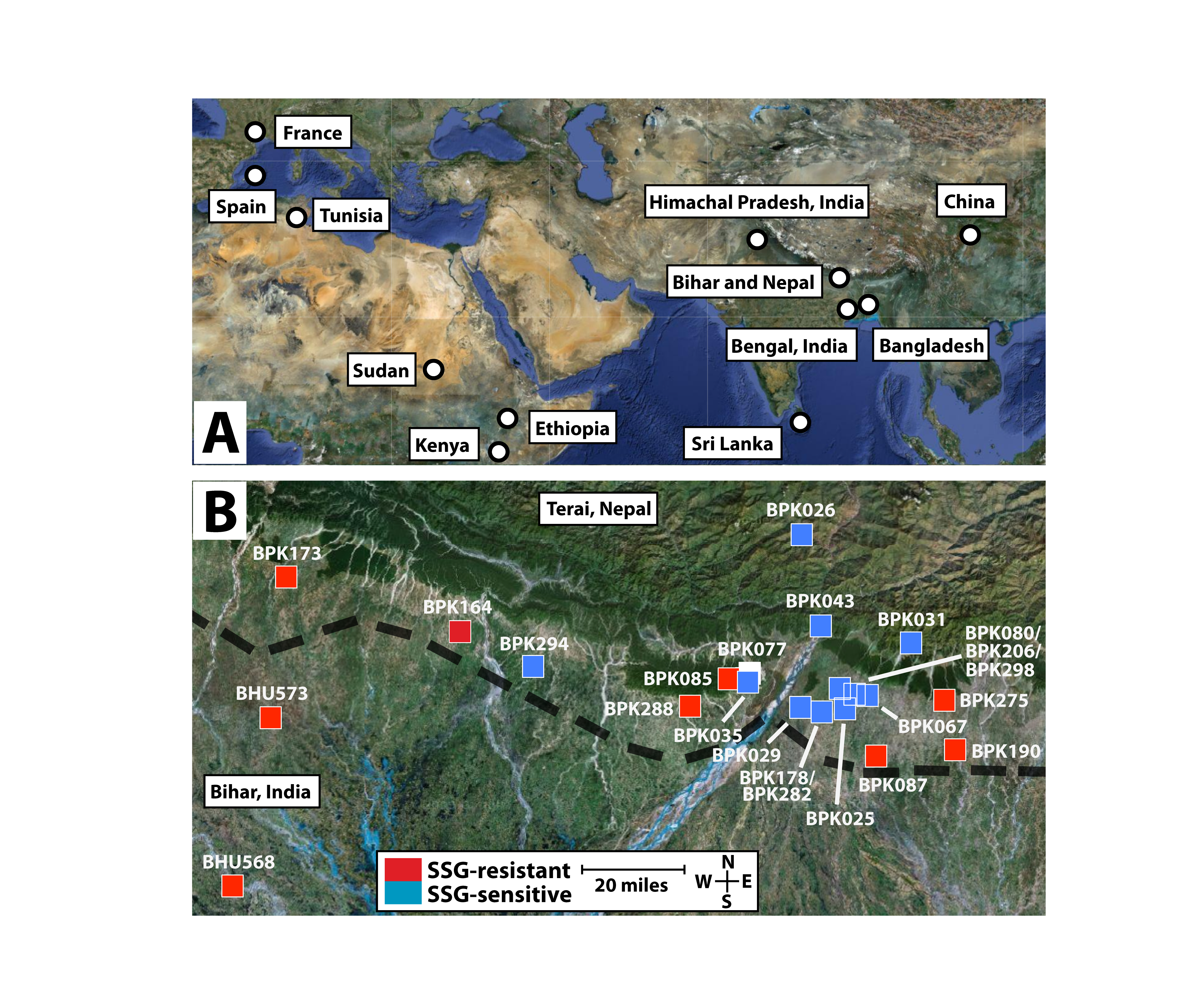


The geographic locations of *L. donovani* species complex studied here shown for: (A) 193 *L. donovani* complex strains from geographically distinct regions: Nepal (52 from Terai), India (93 from Himachal Pradesh, Bihar and Bengal), Bangladesh (21), Kenya (5), Sudan (6), Ethiopia (2), France (3), Sri Lanka (2), Spain (1), Tunisia (1) and China (3) (Supplementary Table 1); and (B) 23 sampled from Nepal and 2 from the Indian state of Bihar – the black line represents the border between the countries. The two most divergent samples (BPK026/0cl5 and BPK031/0cl12) were taken from upland regions with less endemic VL (Bhattarai et al. 2010). SSG-resistant (red) and -sensitive (blue) samples are shown. Although the geographic range was small, there were considerable differences between phenotypes during *in vitro* SSG treatment.

Supplementary Figure 2. Estimating the population structure in 193 strains of the *L. donovani* complex using the clustering approach implemented in Structure.


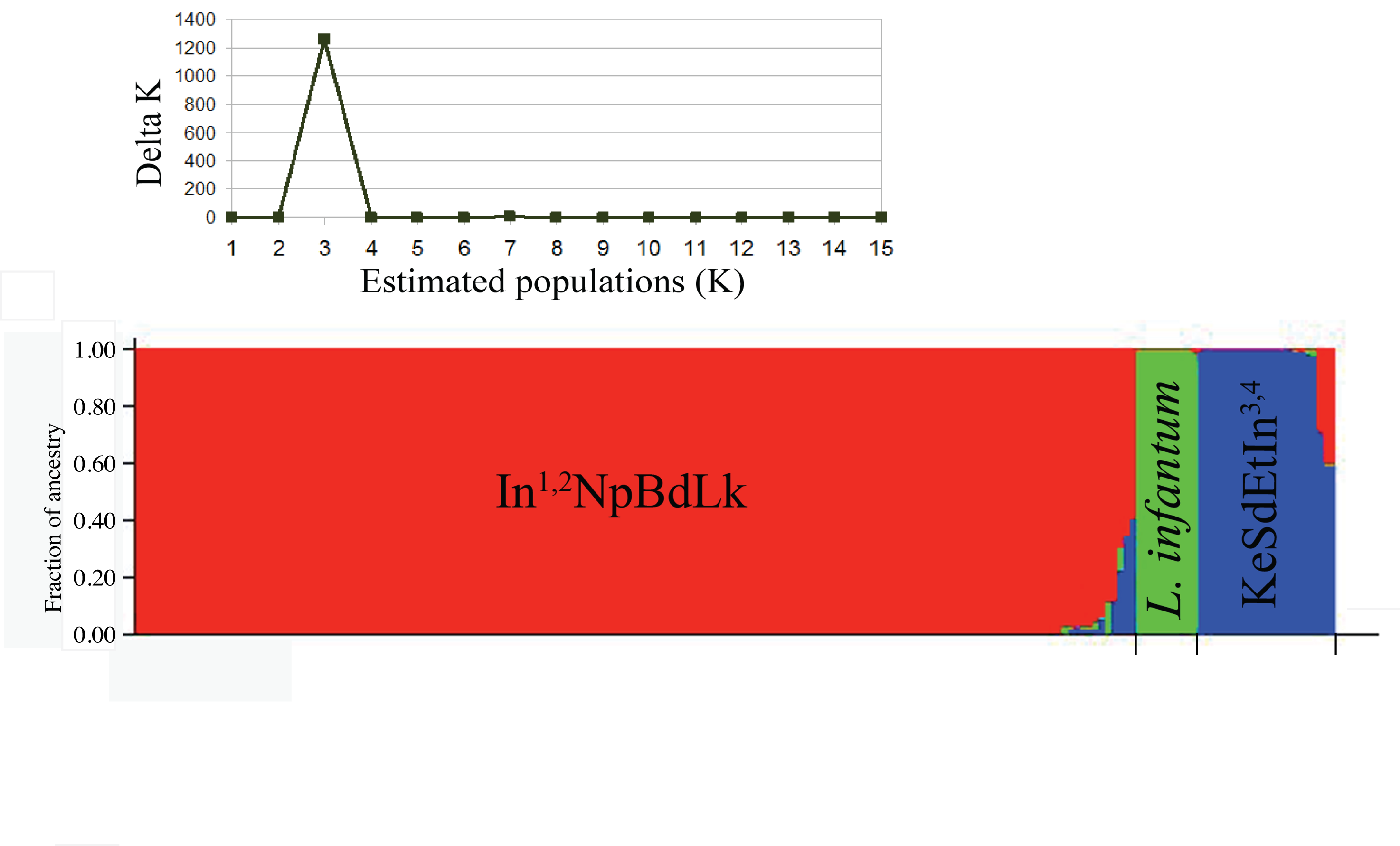


The likely number of populations was determined by Structure clustering for K populations for 193 strains: on the y-axis Delta K values (∆K, Evanno et al. 2005) are shown for given values of K, highlighting that K=3 was the most likely value. Below are the Structure assignation probabilities for each strain to the three different populations, In^1,2^NpBdLk (red: India^1,2^, Nepal, Bangladesh, and Sri Lanka), *L. infantum* (green: China, Spain, France, Tunisia) and KeSdEtIn^3,4^ (blue: Kenya, Sudan, Ethiopia and India^3,4^).

Supplementary Figure 3. Geographical population assignation of 193 strains from *L.*
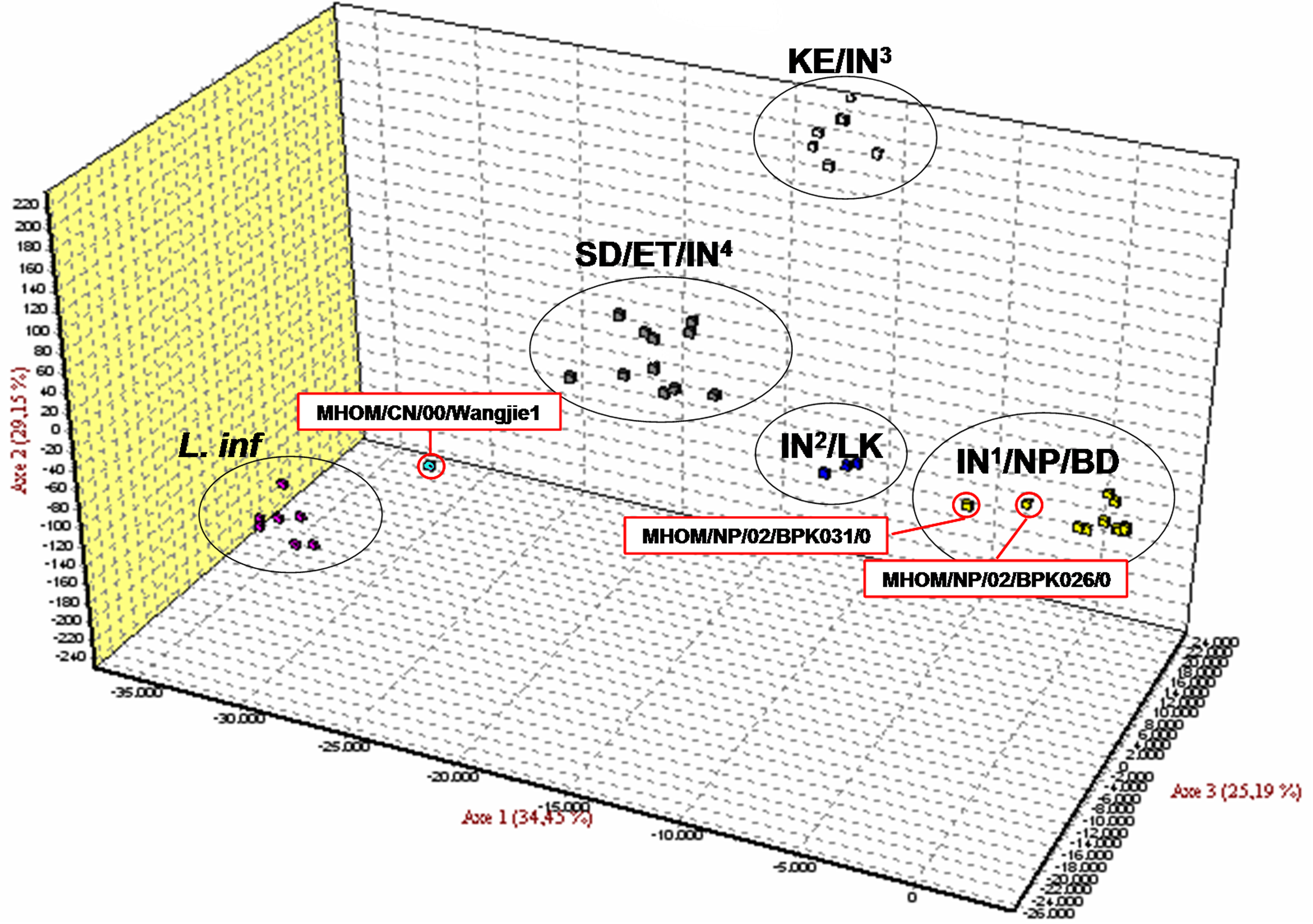
*donovani* complex using microsatellites.

|  |
| --- |
| A three-dimensional factorial correspondence analysis (FCA) illustrated the extent of MLMT diversity in this set of strains from the Indian sub-continent compared to for 25 geographically distant divergent strains using Genetix (v4.05, Belkhir et al. 2002) with data generated from genetic clusters from Structure (K=5; Evanno et al. 2005). This showed the five populations called IN^1^/NP/BD (yellow), IN^2^/LK (blue, including *L. donovani* from Chandigarh from Northeast India and two Sri Lankan strains), KE/IN^3^ (white, with LRC-L51a, LRC-L51p and SC23 from India), SD/ET/IN^4^ (grey, including L13 from India) and *L. infantum* (mauve, representing strains from China, France, Spain and Tunisia). Notably cluster IN^1^/NP/BD was composed of the vast majority of strains (N=161) that were closely related to each other in comparison with the other populations that showed greater variability. BPK026/0cl5 and BPK031/0cl2 were the only Nepalese strains that showed high levels of polymorhism. The position of strain MHOM/CN/2000/Wangjie1 (highlighted) was notable. It was previously identified as *L. donovani*: here it was placed at an intermediate position between *L. infantum* and *L. donovani*, both of which are present in China. The axes plotted display 89% of the total variation (34.5%, 29.2% and 25.2%). |


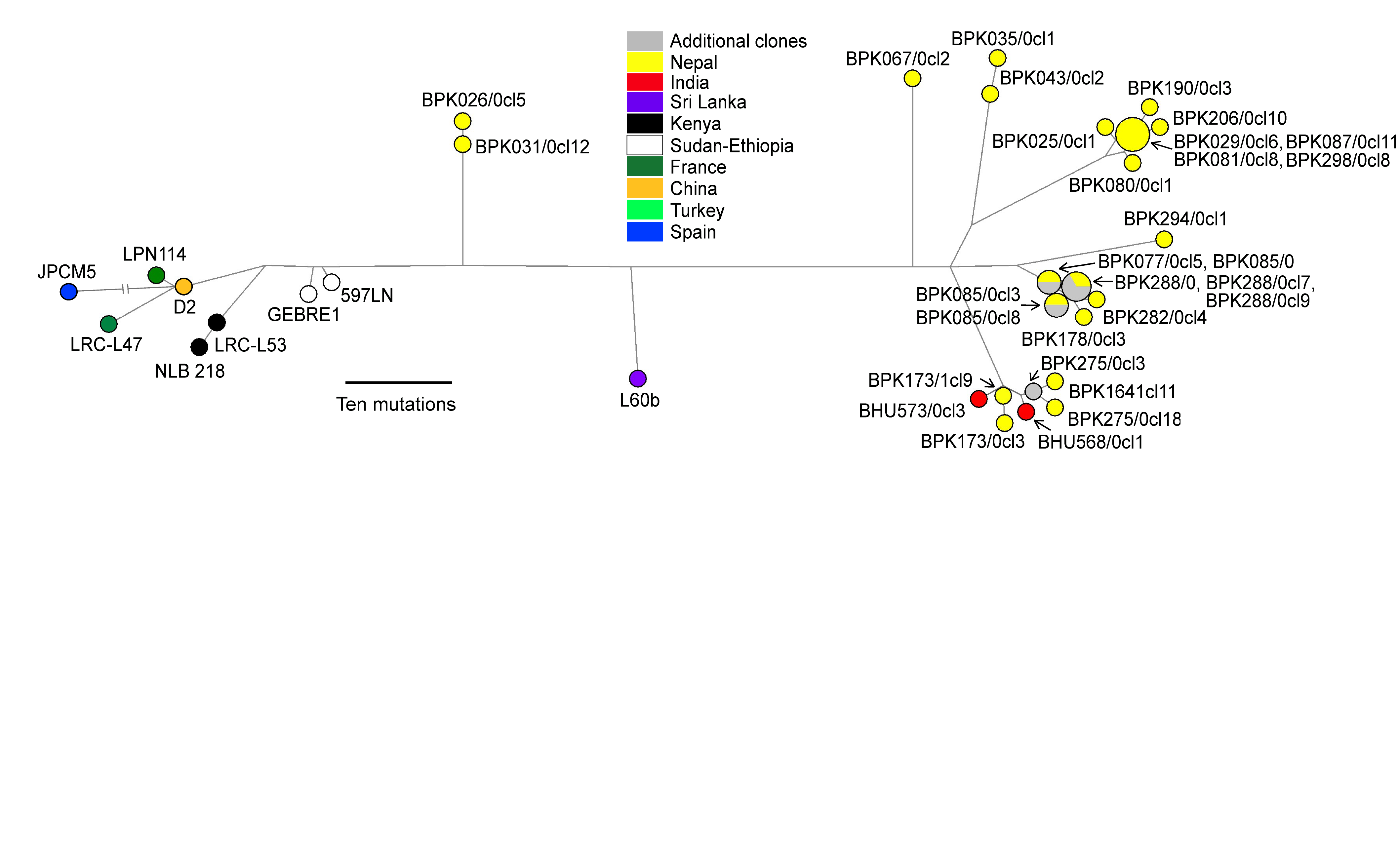


A total of 83% of the total genetic variation between strains was accounted for by the first principle component (PC) reflecting the separation of the Nepal-Bihar samples from the other samples (except BPK026/0cl5 and BPK031/0cl12) in this median joining phylogenetic network constructed with Network v4.2.0.1 (Bandelt et al. 1999). The “Additional clones” represent sub-cloones of the original cloned smaple. The phylogenetic position for the *L. infantum* JPCM5 was estimated using the reference genome (www.genedb.org).

Supplementary Figure 4. SNP-based phylogenetic network of samples from the *L. donovani* complex.


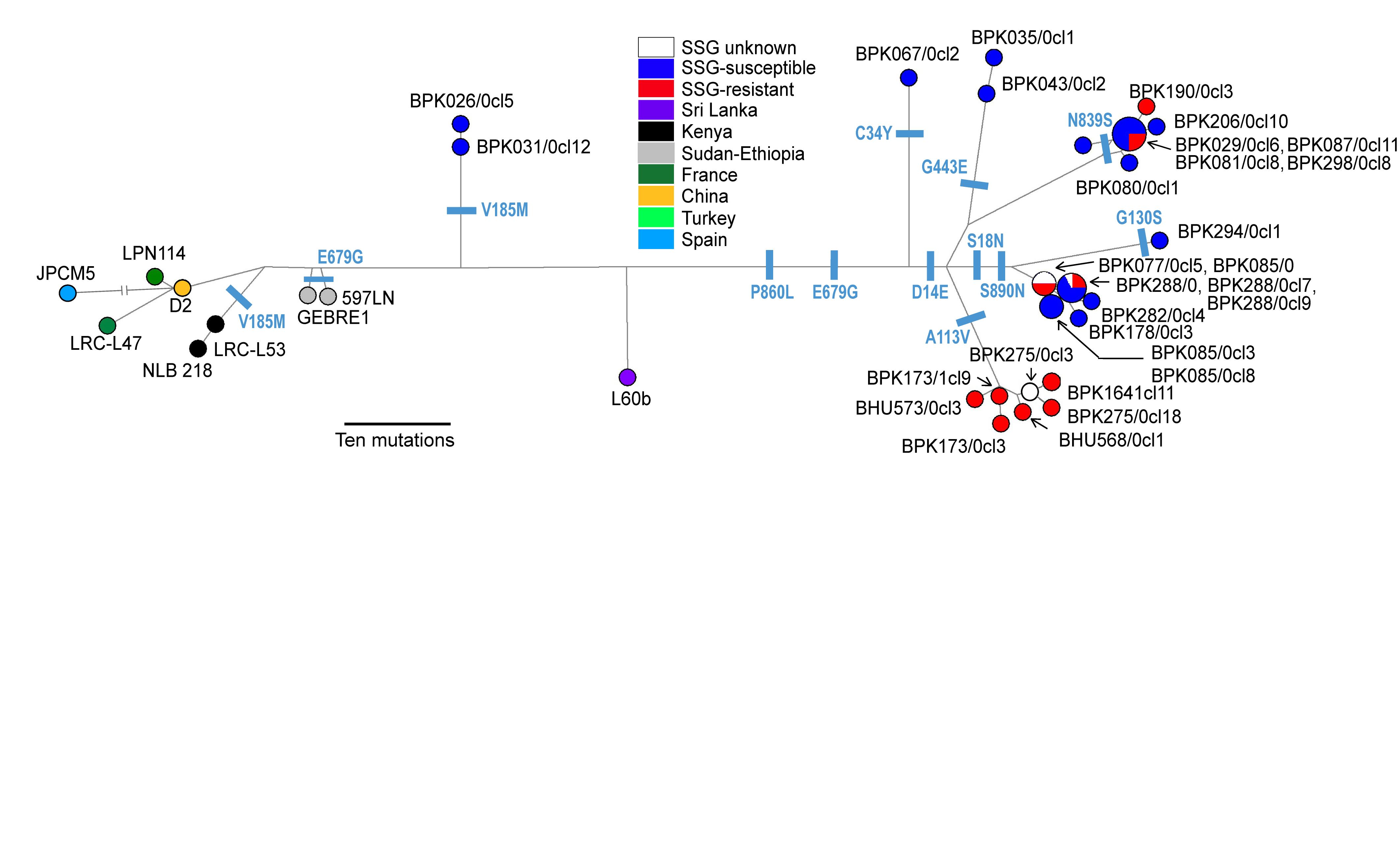


The responses to *in vitro* SSG exposure were classed as resistant (red) or sensitive (blue) where determined for the Nepalese and Bihari lines. Homozygous protein-level SNPs between groups are shown (blue) - see Table 2 for locus information.

Supplementary Figure 5. Phylogenetic network of samples from the *L. donovani* complex with *in vitro* SSG phenotypes indicated.

Supplementary Figure 6. Posterior distribution estimates of the historical change in effective population size in *L. donovani* complex strains.


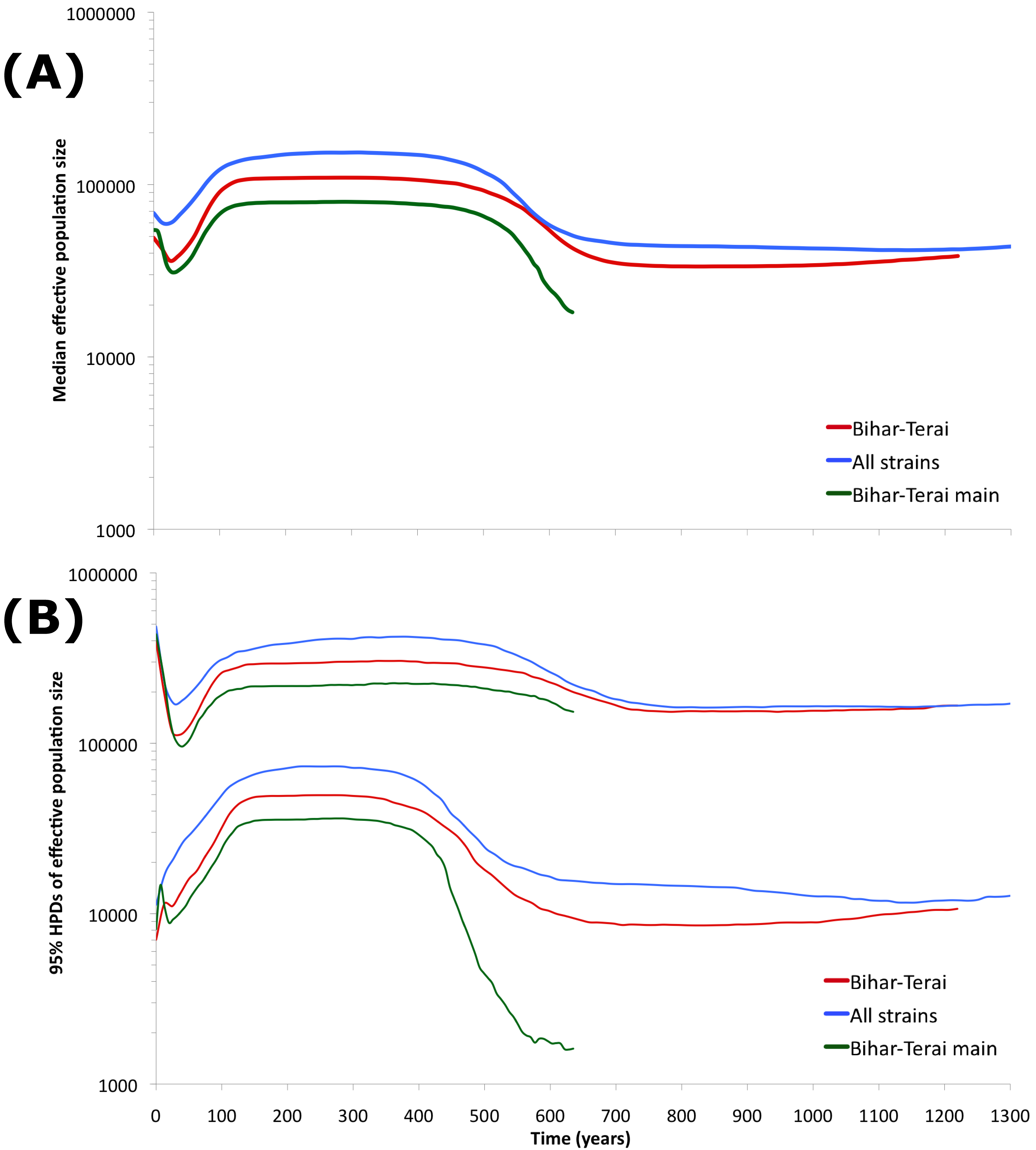


(A) Median and (B) 95% HPDs (highest posterior density) Bayesian skyline posterior distribution estimates of the effective population size (N_e_, log-scaled here) over time (years). Three datasets were simulated independently: the Bihar-Terai set (red) was composed of all strains from Bihar and Terai (25); all strains (blue) were all 33 studied here; and Bihar-Terai main (green) was all strains from Bihar and Terai except divergent clones BPK026/0cl5 and BPK031/0cl12. A mutation rate of 10^-6^ N_e_ and diploidy were assumed to calculate the effective population size from 2N_e_µ, and time was calibrated using a generation time of one day from the *L. infantum* – *L. chagasi* split (Leblois et al. 2011). The bottleneck peak (lowest N_e_) of the Nepalese sample (isolated in 2002-03) coinciding to a large extent with the end of the 1960s DDT spray campaigns in the Indian subcontinent (1974-84 with 95% limits), though HPDs should be the primary focus for confident N_e_ fluctuations inference.


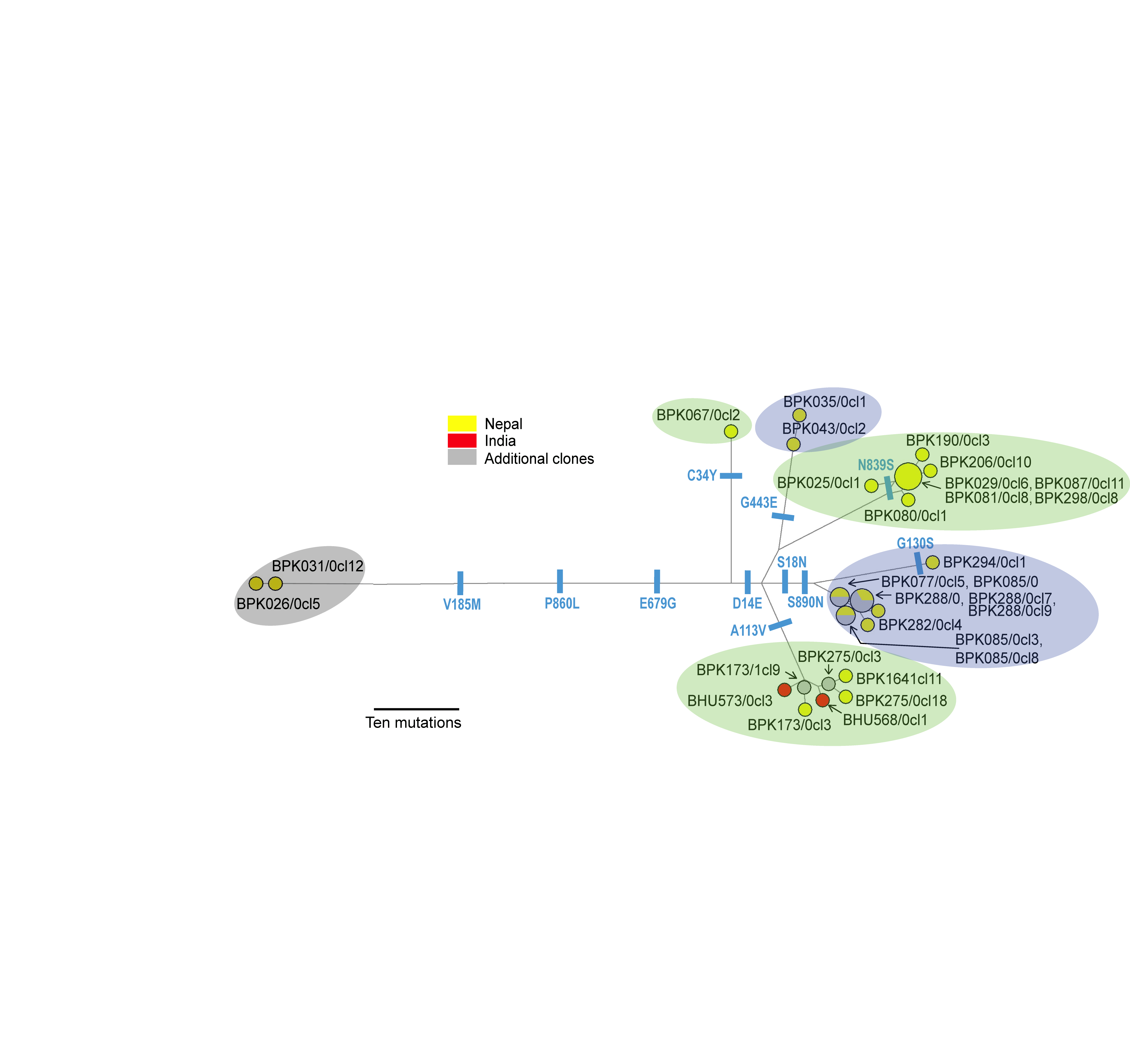


Supplementary Figure 7. Phylogenetic network of Bihari and Nepalsese *L. donovani* lines with geographic origin and kDNA groups indicated.

The colours of the circled groups correspond to different kDNA genotypes class by Bhattari et al. (2010): the first in pale blue containing only BPK026 and BPK031; the second in pale green; and the third in pale grey. The differential clustering of SNP and kDNA markers indicates recombination events may have occurred: the most parsimonious explanation being that these have happened for strains BPK035/0cl1 and BPK043/0cl2 (and perhaps the lineage represented by BPK067/0cl2). The additional clones represent sub-cloones of the original cloned sample.
